# DUF-721 and N-terminal extension of the helicase loader DciA bind ssDNA to promote replicative DnaB helicase loading in *Caulobacter crescentus*

**DOI:** 10.1016/j.jbc.2025.110724

**Published:** 2025-09-15

**Authors:** Keito Watanabe, Shohei Sato, Naoto Itani, Dengyu Wang, Nanato Kiyohara, Satoshi Matsuoka, Tsutomu Katayama, Shogo Ozaki

**Affiliations:** 1 Department of Molecular Biology, Graduate School of Pharmaceutical Sciences, Kyushu University, Higashi-ku, Fukuoka, Japan; 2 Department of Biochemistry and Molecular Biology, Graduate School of Science and Engineering, Saitama University, Saitama-Shi, Saitama, Japan

**Keywords:** DNA replication, DnaB helicase, DNA-protein interaction, protein-protein interaction, DciA

## Abstract

The establishment of replication forks relies on a dedicated molecular system by which a ring-shaped replicative DNA helicase is loaded onto the chromosome DNA, facilitating the unwinding of duplex DNA into single-stranded DNA. In most bacteria, the DnaB helicase coevolved with its accessory protein DciA loader, a member of the domain of unknown function (DUF)-721-containing protein family. In the model bacterium *Caulobacter crescentus*, DciA promotes helicase loading and the C-terminal extension of DUF-721 serves as a specific binding site for the cognate DnaB helicase. However, the mechanistic role of DUF-721 in DnaB helicase loading remains unknown. Here, we provide evidence that the ssDNA binding activity of DUF-721 is crucial for DnaB helicase loading in *C. crescentus*. Using plasmid complementation assays, we identified DciA Arg106 and Leu119 in DUF-721 as essential residues for *in vivo* DciA functions. Biochemical analyses revealed that both residues are essential for helicase loading *in vitro.* Specifically, Arg106 is important for ssDNA binding, with this activity being supported directly or indirectly by Leu119. Yet, these residues are dispensable for DnaB binding. In addition, we reveal the N-terminal extension of DUE-721 is crucial for ssDNA binding and helicase loading. Given the conservation of Arg106 and Leu119 among DciA family proteins, these results suggest that ssDNA binding *via* DciA DUF-721 domain plays a specific and conserved role in helicase loading. Providing a molecular insight into how DciA stimulates helicase loading, our findings highlight a conserved mechanism of ssDNA binding among DciA-family proteins.

During chromosomal replication, replication forks are generated by the action of replicative DNA helicases that unwind double-stranded (ds) DNA into single-stranded (ss) DNA (1, 2, 3). Replicative DNA helicases generally form a ring-shaped oligomer that encircles ssDNA (3). Translocating directionally along the chromosome DNA, the helicase drives the replication fork and allows DNA polymerases to synthesize the complementary strands. In eubacteria, the replicative helicase comprises a homo-hexameric ring of the ubiquitous DnaB-family protein. Fueled by ATP hydrolysis, DnaB helicase translocates along ssDNA in the 5′ to 3′ direction (4, 5, 6, 7). To ensure initiation, the actions of DnaB helicase are regulated by helicase loaders (8, 9). However, in diverse bacterial organisms, the underlying molecular mechanisms of how the helicase loaders introduce the DnaB helicase to ssDNA show diversity and its major mechanism remains elusive.

One of the best-characterized helicase loaders in eubacteria is *Escherichia coli* DnaC (Fig. 1). DnaC, a member of the AAA+ ATPase family, binds to the *E. coli* DnaB (*ec*DnaB) hexamer in a 1:1 ratio, forming the *ec*DnaB-DnaC hetero-complex (6, 9, 10, 11). As a common feature of DnaB-family proteins, the *ec*DnaB protein comprises three functional domains: the N-terminal domain (NTD), the central linker helix domain (LH), and the C-terminal RecA-fold ATPase domain (CTD) (Fig. 2*A*). Of these, the α9 and α14 helices from the LH and CTD, respectively, include specific sites for interaction with the DnaC loader (6, 10). Although the *ec*DnaB hexameric ring is essentially closed in the absence of DnaC, the *ec*DnaB-DnaC complex undergoes a conformational change to form an open-spiral structure competent to engage ssDNA (Fig. 1). Subsequent dissociation of DnaC from the complex allows topological ssDNA loading of the *ec*DnaB hexamer and subsequent translocation of the ssDNA-loaded *ec*DnaB hexamer along the DNA (Fig. 1) (12, 13). Cryogenic electron microscopy and crystal structure analyses demonstrate that the DnaC N terminus specifically interacts with the specific LH and CTD regions which comprise interaction interface of DnaB protomers of the hexameric DnaB ring, imposing distortions on the closed DnaB ring to trigger the open-spiral conformation (10). Although the counterpart of DnaC is conserved as DnaI in *Bacillus subtilis* (14, 15, 16), most bacterial species lack a DnaC/DnaI homolog, and would instead rely on the domestication of DciA-family helicase loaders (16, 17, 18, 19, 20). Thus, elucidation of DciA is important to understand the major mechanism of DnaB loading in the bacterial domain.

**Figure 1:**
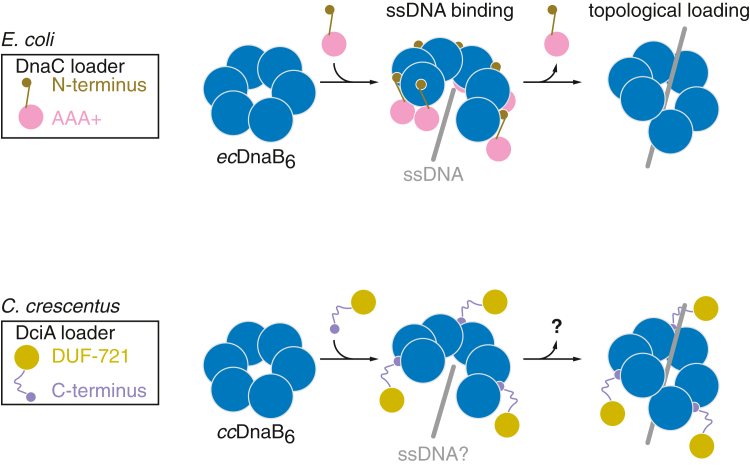
**A model for topological helicase loading.** Hexameric *ec*DnaB (*ec*DnaB_6_) and *cc*DnaB (*cc*DnaB_6_) adopt a closed ring-like conformation in their default state. Upon binding to their respective loader proteins (DnaC for *ec*DnaB and DciA for *cc*DnaB), the hexamers switch to the open-spiral conformation, allowing ssDNA entry in the central channel. Unlike DnaC, which dissociates after topological *ec*DnaB_6_ loading onto ssDNA, DciA may remain associated with DNA-loaded *cc*DnaB_6_.

**Figure 2:**
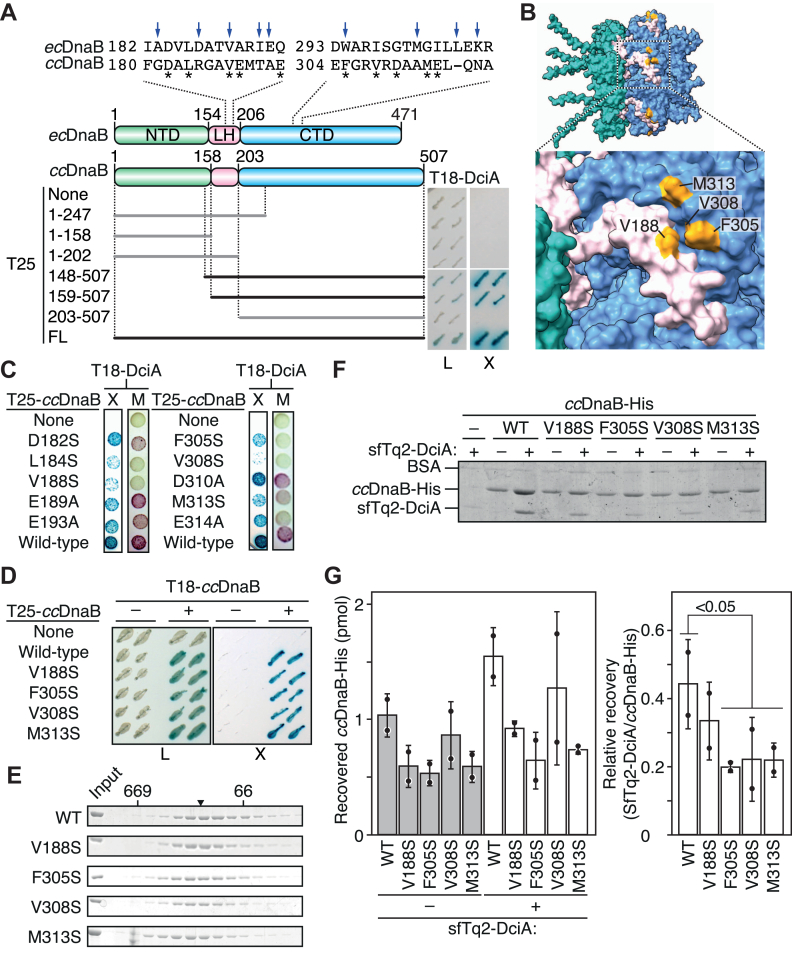
**Determination of *cc*DnaB residues crucial for DciA binding.***A*, bacterial two-hybrid assay. Schematic representations of the N-terminal domain (NTD), linker helix (LH), and C-terminal domain (CTD) of *cc*DnaB and *ec*DnaB are shown. The *Escherichia coli* strain BTH101 was cotransformed with a prey plasmid encoding the T18-fused DciA and a set of bait plasmids encoding T25 fragment fused to full-length *cc*DnaB (FL), truncated versions of *cc*DnaB (1–127, 1–158, 1–202, 148–507, 159–507, 203–507 aa) or no insert (none). Two representative clones for each transformant were assessed for the formation of blue colonies on M63 agar supplemented 0.2% maltose (X) and 40 μg/ml X-gal, 100 μg/ml ampicillin, and 50 μg/ml kanamycin at 30 ˚C for 4 days. *Black* and *gray* bars indicate truncated constructs with positive and negative interaction scores, respectively. As control, all transformants grew normally at 30 ˚C for 2 days on LB agar supplemented 40 μg/ml X-gal, 100 μg/ml ampicillin, and 50 μg/ml kanamycin (L). *Arrows* indicate *ec*DnaB residues responsible for DnaC binding. *Asterisks* indicate *cc*DnaB residues analyzed in *panel C*. *B*, AlphaFold 3-predicted structural model of the *cc*DnaB hexamer. The *cc*DnaB residues Val188, Phe305, Val308, and Met313 highlighted in *orange*, are exposed on the predicted protein surface. *C* and *D*, bacterial two-hybrid analysis of single-amino acid substitution variants of *cc*DnaB. The *Escherichia coli* strain BTH101, cotransformed with a prey plasmid encoding T18-DciA (*C*) or T-18ccDnaB (*D*) and a bait plasmid encoding indicated T25-*cc*DnaB variants, were assessed for blue colony formation at 30 ˚C for 4 days on M63 agar supplemented 0.2% maltose, 40 μg/ml X-gal, 100 μg/ml ampicillin, and 50 μg/ml kanamycin (X) or red colony formation at 30 ˚C for 2 days on MacConkey agar supplemented with 2% maltose, 100 μg/ml ampicillin and 50 μg/ml kanamycin (M). *E*, size-exclusion chromatography. Wild-type DnaB-His (WT) or DnaB-His (V188S, F305S, V308S, and M313S) were subjected to a Superdex 200 column. The elution fractions were analyzed by SDS-14% PAGE and Coomassie brilliant blue staining. The elution peaks of molecular weight marker proteins (660 kDa thyroglobulin and 66 kDa bovine serum albumin) are indicated. An *arrowhead* marks the predicted elution peak position for a globular protein with the size of 330 kDa, corresponding to a DnaB-His hexamer. *F* and *G*, pull-down assay. Wild-type DnaB-His (WT) or its variants (480 nM) were incubated in the presence (+) or absence (−) of sfTq2-DciA (850 nM), followed by pull-down using Ni-conjugated magnetic beads. After washing, the materials retained on the beads were analyzed using SDS-14% PAGE and Coomassie brilliant *blue* staining. A representative gel (*F*) and bar graphs (*G*) with scatter plots generated from two independent reactions are shown. The *p* value was calculated using Student's *t* test. sfTq2-DciA, superfolder mTurquoise2 fluorescent protein-tagged DciA.

DciA family proteins are widely conserved in the bacteria domain and are demonstrated to be essential for chromosomal replication in diverse model organisms including *Caulobacter crescentus*, *Pseudomonas aeruginosa*, *Mycobacterium tuberculosis*, and *Vibrio cholerae* (17, 18, 20, 21, 22, 23). Biochemical characterizations suggest that DciA homologs directly interact with their cognate DnaB helicases to facilitate topological helicase loading. In *C. crescentus* DciA, a predicted short α-helix at the C terminus includes conserved leucine residues responsible for binding to *C. crescentus* DnaB (*cc*DnaB) and is essential for DciA *in vivo* functions (18) (Fig. 1). The functional importance of this C-terminal region is further highlighted by studies on the *V. cholerae* DciA homolog, in which a truncated *V. cholerae* DciA variant lacking the C-terminal region showed reduced binding affinity for its cognate DnaB protein (24). Consistently, in the recent crystal structure of *V. cholerae* DciA-DnaB complexes, the C-terminal α-helix of *V. cholerae* DciA was positioned near the α9 and α14 helices of *V. cholerae* DnaB, which correspond to the DnaC binding helices in *E. coli* DnaB (25). These findings suggest that, despite structural differences, the core interactions between helicase and loader are conserved in the DnaB-DciA and DnaB-DnaC systems. However, the specific DnaB residues responsible for DciA binding as well as mechanisms in ssDNA interaction for topological DnaB loading by DciA remain to be elucidated.

The C-terminal region of *C. crescentus* DciA is connected to a distinct structural scaffold termed DUF (domain of unknown function)-721 *via* an unstructured linker (16) (Fig. 1). Despite substantial sequence diversity, DUF-721 is structurally predicted to resemble the K homology (KH) domain (pfam: PF07650). Proteins containing KH domains typically bind oligonucleotides such as ssRNA and ssDNA and participate in diverse cellular activities, including transcriptional and translational regulation, in both prokaryotes and eukaryotes (23, 26). Consistent with this, *in vitro* DNA binding assays have shown that mycobacterial DciA and *V. cholerae* DciA can interact with dsDNA or ssDNA (20, 24). Furthermore, *V. cholerae* DciA undergoes liquid–liquid phase separation in the presence of ssDNA *in vitro*, supporting the view that DciA is an ssDNA binding protein. Mutant analyses identified Arg2, Arg5, and Lys26 resides within the N-terminal region connecting to DUF-721 of *V. cholerae* DciA, as being directly or indirectly involved in ssDNA binding (24). Notably, however, none of these residues are critical for the helicase activity of *V. cholerae* DnaB *in vitro*. Therefore, the exact role of DUF-721 and the biological significance of the ssDNA binding activity in the DciA family proteins remain to be elucidated.

In this study, we aim to elucidate the mechanism by which DciA stimulates helicase loading in *C. crescentus,* focusing on interactions with DnaB and ssDNA interaction. Using a bacterial two-hybrid system and biochemical assays, we demonstrate that specific hydrophobic residues in the α9 and α14 helices within the LH and CTD domains of ccDnaB are prerequisite for DciA binding. Moreover, to investigate the role of the conserved DUF-721 domain in *C. crescentus* DciA, we conducted plasmid complementation tests and identified Arg106 and Leu119 as essential residues for *in vivo* DciA functions. Biochemical characterizations further reveal that these residues are important for ssDNA binding of DciA and for topological loading of *cc*DnaB helicase *in vitro*. Our mutant analysis also suggests that the N-terminal extension of DUE-721 is crucial for ssDNA binding and helicase loading. Since neither these residues nor the N-terminal extension is required for *cc*DnaB-DciA binding, our findings suggest that ssDNA binding by DciA plays a specific role in simulating topological helicase loading. Given the conservation of the Arg106 and Leu119 residues among DciA family proteins, we propose that the role of ssDNA binding by DciA represents a common mechanism in bacterial helicase loading.

## Result

### *E. coli* and *C. crescentus* DnaB proteins share the same contact surfaces for interaction with the cognate helicase loader

To determine functional *cc*DnaB structure crucial for DciA interaction in *C. crescentus*, we carried out a bacterial two-hybrid analysis. When we assessed the full-length *cc*DnaB, fused N terminally to T25 fragment of the adenylate cyclase, for its interaction with DciA fused N terminally to the T18 fragment, we readily observed a positive signal: formation of blue colonies on the M63 synthetic minimal medium supplemented with maltose and X-gal (Fig. 2*A*). Similar positive signals were detected between DciA and the truncated *cc*DnaB variants 148 to 507 and 159 to 507, which span the LH-CTD domains. In contrast, the *cc*DnaB variant 1 to 158, bearing only the NTD, scored negative, indicating that the NTD of *cc*DnaB is dispensable for interaction with DciA. Furthermore, negative signals for the *cc*DnaB variants 1 to 202 (bearing NTD and LH) and 203 to 507 (bearing CTD) indicated that neither the LH nor the CTD alone is sufficient for DciA binding. Thus, the contact surfaces of *C. crescentus* DnaB in complex with DciA likely resides in both the LH and CTD domains.

The interaction between *E. coli ec*DnaB and DnaC relies on the specific contact surfaces (the α9 and α14 helices) within the LH and CTD domains of *ec*DnaB. Although these residues are less conserved in *cc*DnaB (Fig. 2*A*), we hypothesized that the corresponding surfaces of *cc*DnaB could bind DciA, with certain residues having diverged during the evolution of each loader. This is consistent with our previous observation of orthogonal interactions between DciA and *ec*DnaB. To test this hypothesis, we carried out bacterial two-hybrid analysis using single amino acid substitutions in selected *cc*DnaB residues (Asp182, Leu184, Val188, Glu189, Glu193, Phe305, Val308, Asp310, Met313, and Glu314), which are exposed on the potential contact surface of a predicted *cc*DnaB structure (Fig. 2, *A* and *B*). As judged by the formation of blue colonies on M63+maltose+X-gal agar plates, the T25-*cc*DnaB variants (L184S and V188S, F305S, V308S, and M313S) moderately inhibited interaction with T18-DciA (X in Fig. 2*C*). Consistently, these variants scored negative in T18-DciA interaction on MacConkey-maltose agar; these variants, as well as T25 alone, formed pale colonies whereas WT T25-*cc*DnaB formed red colonies (M in Fig. 2*C*). Moreover, T25-*cc*DnaB (E314A) yielded an intermediate phenotype, displaying positive on M63+maltose+X-gal agar and negative on MacConkey-maltose agar. Both WT T25-*cc*DnaB and T25-*cc*DnaB variants maintained their ability for DnaB-DnaB interaction, as shown by positive signals with WT T18-*cc*DnaB (Fig. 2*D*). Thus, these findings suggested that *cc*DnaB residues Leu184, Val188, Phe305, Val308, Met313, and Glu314 are directly or indirectly involved in DciA binding. The residual activities of their variants suggest that multiple residues constituting the contact surface for DciA.

To further elucidate the role of these newly identified functional structures, we focused on representative loss-of-function alleles (V188S, F305S, V308S, and M313S). To biochemically characterize the interaction between *cc*DnaB and DciA, we purified recombinant *cc*DnaB proteins with a C-terminal hexahistidine tag (*cc*DnaB-His). Size-exclusion chromatography using a Superdex 200 column revealed that the WT *cc*DnaB-His formed oligomers with an average molecular weight consistent with homo-hexamer formation, as previously reported (18) (Fig. 2*E*). Similar oligomer formation activity was sustained for the *cc*DnaB-His variants (V188S, F305S, V308S, and M313S) (Fig. 2*E*), which is consistent with the bacterial two-hybrid analysis (Fig. 2*D*) and supported the idea that the mutated residues are unlikely to affect the overall structure of the *cc*DnaB hexamer.

Next, we carried out pull-down assays to investigate the direct interaction between *cc*DnaB and DciA using a superfolder mTurquoise2 fluorescent protein-tagged DciA (sfTq2-DciA). The sfTq2 tag improves visibility of the recovered DciA (18). When WT *cc*DnaB-His was coincubated with sfTq2-DciA, coelution of the two was observed, as previously reported (18) (Fig. 2, *F* and *G*). Notably, we found that the *cc*DnaB-His variants (V188S, F305S, V308S, and M313S) significantly reduced the affinity for sfTq2-DciA (Figs. 2, *F* and *G*, S1*A*). These data suggested that *cc*DnaB Val188, Phe305, Val308, and Met313 play specific roles in DciA binding. Given specific leucine residues within the C terminus of DciA constitute a binding site for *cc*DnaB, a hydrophobic interaction likely underlies the DciA-*cc*DnaB interaction. Because these *cc*DnaB residues are well conserved at corresponding positions of *V. cholerae* DnaB (Leu187, Trp291, Ile294, and Met302) (Fig. S1*B*), the hydrophobic interaction could be a common mechanism.

### Determination of the functional DciA residues within DUF-721

**Figure 3:**
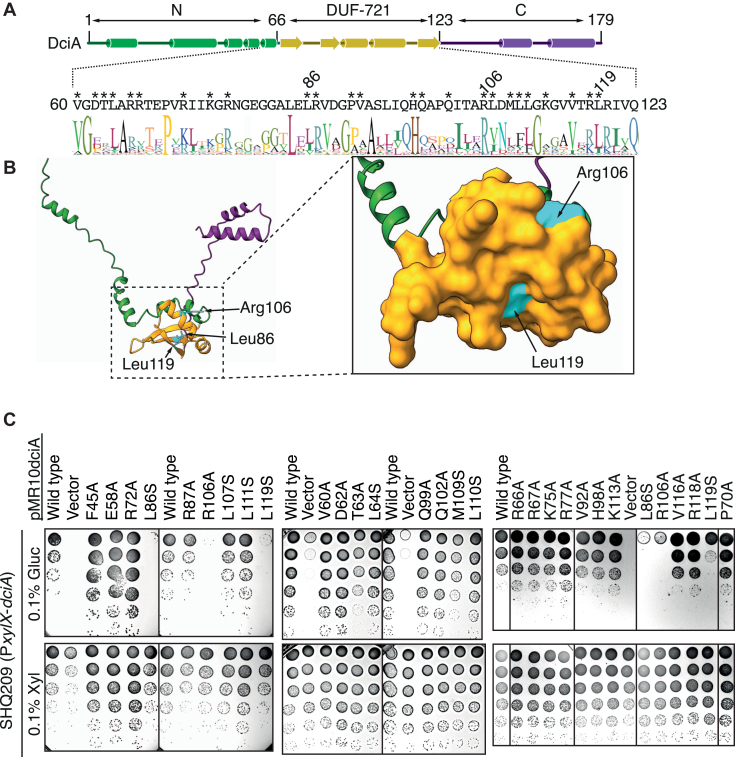
**The DciA DUF-721 is essential *in vivo*.***A*, multiple sequence alignments of DUF-721 were created by Protein BLAST search for DciA and the top 500 sequence data were used to generate Weblogo representation. The schematic shows the predicted secondary structures (*cylinders*, alpha helices; *arrows*, beta strands). Each domain (N, DUF-721, C) is highlighted in different colors. *Asterisks* indicate DciA residues analyzed in *panel C*. *B*, AlphaFold2-predicted structural model of DciA. The DciA Leu86, Arg106, and Leu119 residues are highlighted. The Leu86 residue is buried and not visible in the surface representation. *C*, plasmid complementation test. The SHQ209 strain harboring pMR10 (vector) or pMR10dciA derivatives with the indicated *dciA* allele were grown overnight at 30 ˚C in PYE medium supplemented with 0.1% xylose. Five-fold serial dilutions of the overnight culture were spotted on PYE agar supplemented with kanamycin and 0.1% xylose or 0.1% glucose, followed by incubation at 37 ˚C or 2 days. DUF, domain of unknown function.

Next, we focused on investigating the mechanistic role of the DNAL domain in DciA. Despite its ubiquity among DciA homologs, DUF-721 of *C. crescentus* DciA is dispensable for *cc*DnaB binding, raising the critical question of how this domain contributes to helicase loading. To address this, we first probed for residues within this domain that are essential for DciA functions *in vivo*. Based on a predicted DciA structure (Fig. 3, *A* and *B*), we selected residues that are likely exposed on the protein surface and assessed their roles in DciA activity *in vivo* using a plasmid complementation assay. The host strain (SHQ209) used in this assay integrates the xylose-dependent promoter (P*xylX*) flanked by the *E. coli rrnB* T1T2 transcriptional terminator (*rrnB_ter*) into the *dciA* promoter locus (18). Consistent with the essentiality of DciA, colony formation in this strain was severely compromised in the absence of the inducer, xylose (Fig. 3*C*). These defects were restored by introducing a plasmid bearing the WT *dciA* allele, but not an empty vector (Fig. 3*C*). Single amino acid substitution analyses on plasmid-borne DciA revealed that the *dciA* alleles *L86S*, *R106A*, and *L119S* failed to support colony formation in DciA-depleted cells. Western blotting analyses suggested these alleles did not alter DciA protein stability (Fig. S2), supporting the idea that DciA residues Leu86, Arg106, and Leu119 play specific roles in *cc*DnaB operation.

### DciA Arg106 and Leu119 are dispensable for *cc*DnaB binding

**Figure 4:**
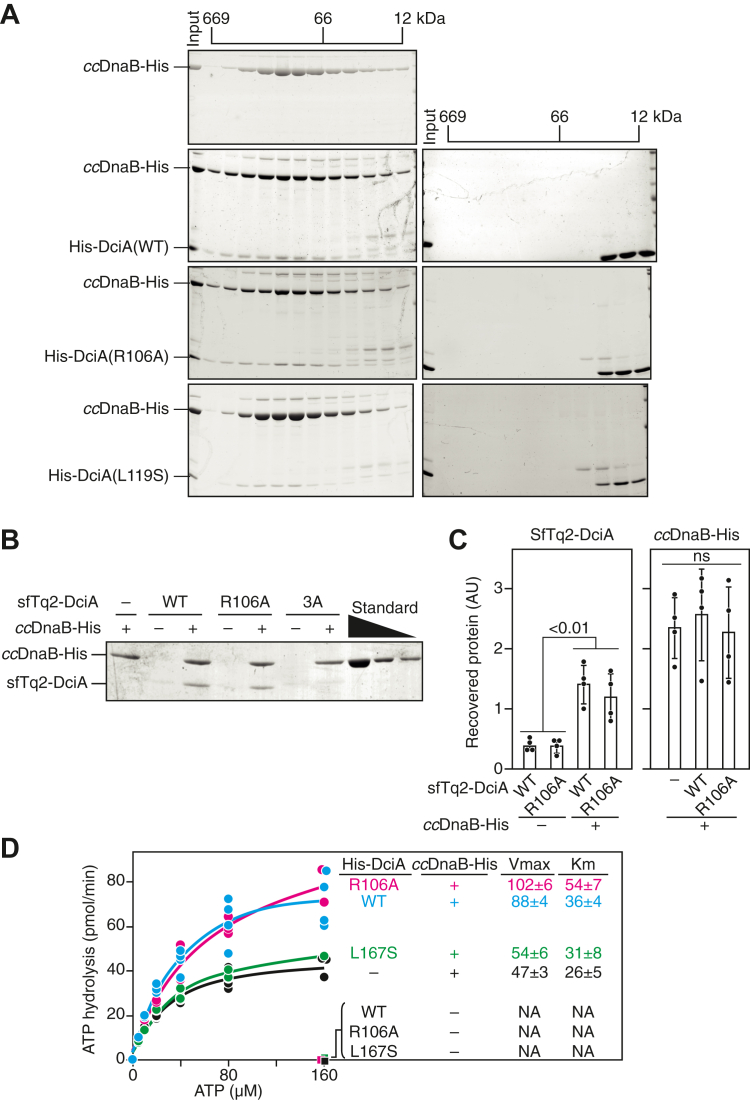
**DciA Arg106 and Leu119 are dispensable for *cc*DnaB binding.***A*, size-exclusion chromatography. *cc*DnaB-His (2.6 nmol), His-DciA (1.3 nmol) or a mixture of both were separated using a Superdex 200 column. Elution fractions were analyzed by SDS-12% PAGE and Coomassie brilliant blue staining. The elution peaks of molecular weight marker proteins (660 kDa thyroglobulin, 66 kDa bovine serum albumin, 12 kDa cytochrome C) are indicated. Samples containing ccDnaB-His are shown on the *left* while those without ccDnaB-His are shown on the *right*. His-DciA (WT) samples are shown in the second gels; His-DciA (R106A) and His-DciA (L119S) samples are shown on the third and bottom gels, respectively. *B* and *C*, pull-down assay. WT sfTq2-DciA or sfTq2-DciA (R106A) (850 nM) were incubated in the presence (+) or absence (−) of WT DnaB-His followed by pull-down using Ni-conjugated magnetic beads. After washing, materials retained on the beads were analyzed using SDS-14% PAGE and Coomassie brilliant blue staining. A representative gel (*A*) and bar graphs (*B*) with scatter plots generated from three independent reactions are shown. The *p* value was calculated using Student's *t* test. ns, not significant. The triple mutant 3A, sfTq2-DciA (L163S L167S L170S) that mutated three conserved leucine residues at the C terminus of DciA, is used as negative control. *D*, ATPase assay. Wild-type His-DciA or His-DciA (R106A or L167S) (100 nM) were incubated at 30 ˚C for 5 min with DnaB-His (8.3 nM as hexamer; 50 nM as monomer) in the presence of various [α-32P]ATP concentrations (5–160 μM). Conversion of ATP to ADP was quantified by TLC analysis and the ATPase rate was fit using Michaelis–Menten kinetics in JMP statistical software (https://www.jmp.com). The equation is as follows: ab/(x + b), where a, b, and x represent the maximum velocity, the Michaelis constant, respectively, and the ATP concentration, respectively. Individual parameters were shown in Table S1. sfTq2-DciA, superfolder mTurquoise2 fluorescent protein-tagged DciA.

To gain molecular insights into the role of the DciA DUF-721 domain, we purified recombinant *cc*DciA proteins with an N-terminal hexahistidine tag (His-DciA) from *E. coli* overproducers using nickel affinity purification followed by size-exclusion chromatography (SEC). As previously reported (18), WT His-DciA was stable as a monomer in solution and retained affinity for *cc*DnaB-His proteins (Fig. 4*A*). Similar profiles were observed for His-DciA (R106A) and His-DciA (L119S) variants (Fig. 4*A*), indicating that the Arg106 and Leu119 residues of DciA are dispensable for *cc*DnaB binding. In contrast, the His-DciA (L86S) variant was insoluble in *E. coli* and as yielded minimal recovery after nickel affinity purification (data not shown), suggesting that Leu86 is crucial for maintaining the structural integrity of DciA.

Focusing specifically on the DciA Arg106 residue, we conducted two complementary biochemical assays to assess its dispensability in *cc*DnaB binding. First, a pull-down assay using ccDnaB-His and sfTq2-DciA (R106A) demonstrated that similar to the WT sfTq2-DciA, sfTq2-DciA (R106A) variant coeluted with *cc*DnaB-His (Fig. 4, *B* and *C*). In contrast, the triple mutant 3A, sfTq2-DciA (L163S L167S L170S), which harbors L163S, L167S, and L170S substitutions in the conserved C-terminal leucine residues, exhibited markedly reduced affinity for *cc*DnaB-His. Second, we evaluated the ATPase activity of *cc*DnaB-His. Our previous study showed that *cc*DnaB hydrolyzes ATP irrespectively of the presence or absence of DNA (18). Consistently, *cc*DnaB-His hydrolyzed ATP in the absence of ssDNA, which was stimulated by WT His-DciA (Fig. 4*D*). Conversely, the His-DciA (L167S) variant, which impairs affinity for *cc*DnaB, failed to stimulate the ATPase activity of DnaB-His (Fig. 4*D*), indicating that ATP hydrolysis by *cc*DnaB is stimulated through its direct binding to DciA. Analysis of His-DciA (R106A) variant revealed that it stimulated the ATPase activity of *cc*DnaB-His at a level comparable to that of WT His-DciA (Fig. 4*D*), further supporting that His-DciA (R106A) maintains its affinity for *cc*DnaB-His. Together, these results indicate that the essential role of DciA Arg106 is independent of DnaB binding.

### DciA Arg106 is important for ssDNA binding

**Figure 5:**
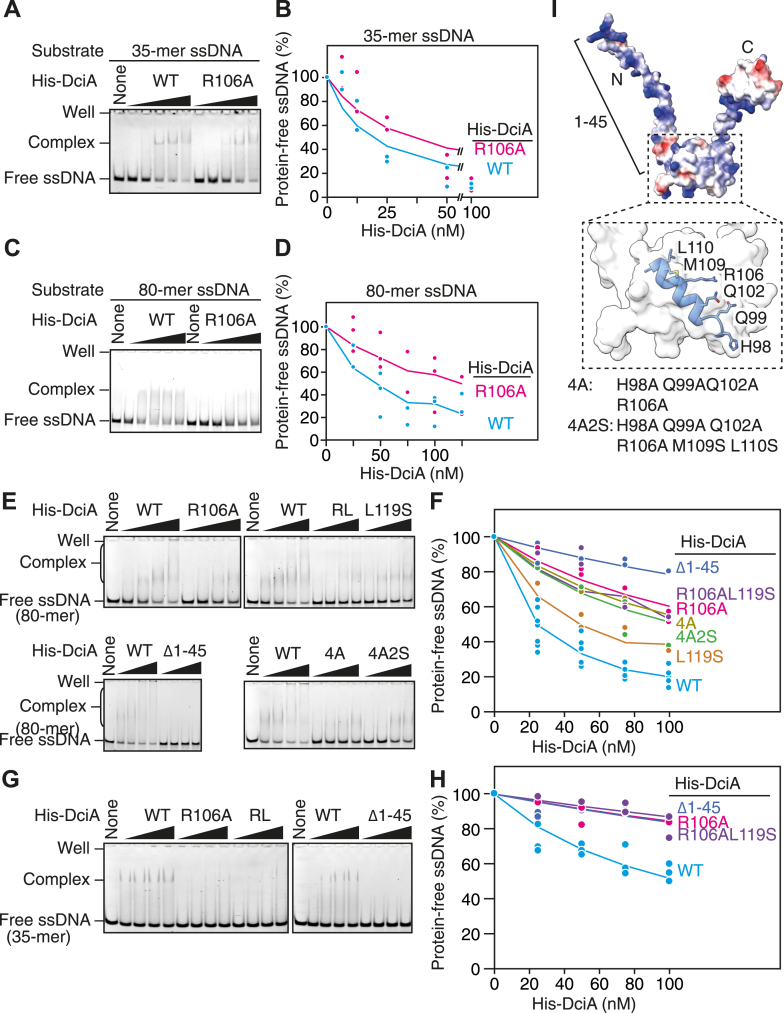
**DciA Arg106 and Leu119 are important for ssDNA binding.***A*–*H*, ssDNA binding assay. WT His-DciA or His-DciA variants were incubated for 5 min with 5′-FAM-labeled ssDNA (35-mer for *A*, *B*, *G*, and *H*; 80-mer for *C*–*F*) in buffer containing 50 mM (*A*–*D*) or 150 mM potassium glutamate (*E*–*H*). Reactions were analyzed using PAGE (6% gels for 80-mer ssDNA and 9% gels for 35-mer ssDNA). Representative gel images are shown (*A*, *C*, *E*, and *G*). The data were analyzed using the Langmuir binding equation: 1-year = x/(Kd+x), where x is the protein concentration (nM), and y is the fraction of protein-free DNA. The Kd values were summarized in Table S2. The average predicted values at each protein concentration were calculated from all fitted curves and connected by lines (*B*, *D*, *F*, and *H*). *I,* surface electrostatic potential of AlphaFold-predicted DciA (*red*: negative potential; *white*: neutral; *blue*: positive potential), visualized using ChimeraX (44). Residues flanking Arg106 (H98, Q99, Q102, M109, and L110) are highlighted. His-DciA (4A) is a quadruplex mutant with H98A, Q99A, Q102A, and M109A substitutions. His-DciA (4A2S) is a derivative of His-DciA (4A) bearing two additional substitutions: M109S and L110S.

Next, we performed an electrophoretic mobility shift assay (EMSA) to investigate whether the DciA Arg106 and Leu119 residues are involved in ssDNA binding (Fig. 5, *A*–*D*). DUF-721 of DciA shares a structural homology with the KH domain (pfam: PF07650), which is found in a wide range of proteins that exhibit affinity for RNA or ssDNA. Previous studies have shown that DciA homologs from *M. tuberculosis* and *V. cholerae* bind ssDNA. Consistent with these findings, our EMSA demonstrated that WT His-DciA binds 30-mer and 80-mer ssDNA substrates in the presence of 50 mM potassium glutamate (Fig. 5, *A*–*D*), suggesting that ssDNA binding is a conserved feature among DciA family proteins. WT His-DciA formed a relatively stable complex with the 30-mer ssDNA substrate, yielding a discrete shifted band (Fig. 5*A*). In contrast, when mixed with 80-mer ssDNA substrates, the shifted-band appeared smear (Fig. 5*A*), indicating that the longer ssDNA may bind multiple DciA molecules. Strikingly, the His-DciA (R106A) variant reduced ssDNA binding compared to WT His-DciA (Fig. 5, *A*–*D*); specifically, for the 30-mer ssDNA, the estimated Kd values using the Langmuir binding equation were 20 ± 7 nM for WT and 36 ± 18 nM for R106A, and for the 80-mer ssDNA, 50 ± 24 nM for WT and 152 ± 75 nM for R106A (Table S2). The residual ssDNA binding activity for His-DciA (R106A) was largely diminished in buffer containing higher salt concentrations (150 mM potassium glutamate) (Fig. 5, *E*–*H*), suggesting that the DciA Arg106 residue is directly or indirectly involved in ssDNA binding. Similarly, the His-DciA (L119S) variant exhibited a reduced affinity for ssDNA under higher salt conditions (Fig. 5, *E*–*H*). The double mutant His-DciA (R106A L119S) also showed compromised ssDNA binding (RL in Fig. 5, *E*–*H*), with a slightly greater impairment than either single mutant. His-DciA (R106A L119S) sustained intact affinity for *cc*DnaB-His, as determined by SEC (Fig. S3). These results suggest that DciA Leu119 directly or indirectly facilitates Arg106-mediated ssDNA binding.

Prompted by the residual ssDNA binding activity of His-DciA (R106A), we speculated that the positively charged N-terminal extension of DciA might contribute to ssDNA binding (Figs. 5*I*, S4, *A* and *B*). To test this idea, we purified His-DciA (Δ1–45), a variant lacking residues 1 to 45. Size-exclusion chromatography showed that the interaction between His-DciA (Δ1–45) and *cc*DnaB-His was substantially sustained. Strikingly, EMSA revealed that His-DciA (Δ1–45) markedly diminished affinity for ssDNA (Fig. 5, *E*–*H*), suggesting that the N-terminal extension is involved in ssDNA binding by DciA.

We also considered the possibility that the residues flanking Arg106 (His98, Gln99, Gln102, Met109, and Leu110), which are likely exposed on the protein surface, may contribute to ssDNA binding (Fig. 5*I*). However, simultaneous substitutions in His-DciA 4A (H98A, Q99A, Q102A, and R106A) and His-DciA 4A2S (H98A, Q99A, Q102A, R106A, M109S, and L110S) did not exacerbate the residual ssDNA binding activity observed in His-DciA (R106A) (Fig. 5, *F* and *G*). Given that each single substitution retained plasmid complementation activity (Fig. 3), we suggest that Arg106 and Leu119 play specific roles within the DUF721 domain of DciA.

### DciA (R106A) and DciA (L119S) sustain stimulation of the DnaB helicase activity

**Figure 6:**
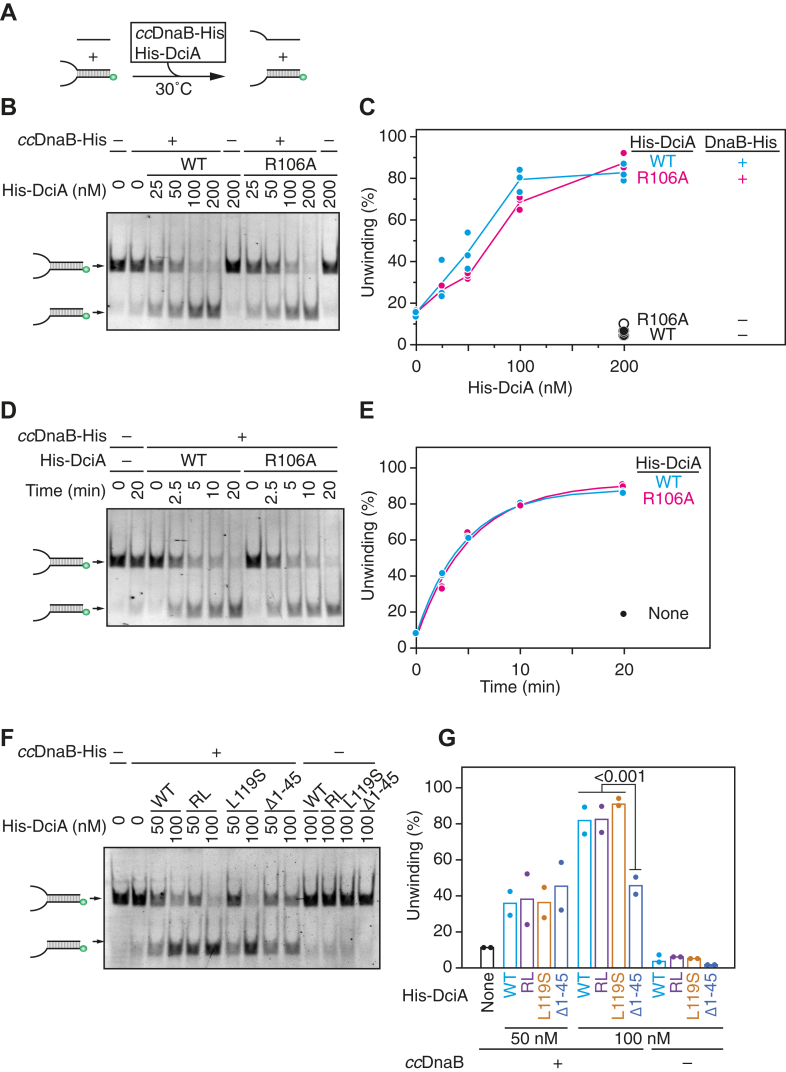
**DciA (R106A) and DciA (L119S) stimulate the *cc*DnaB helicase activity.** The bottom strand of the forked DNA substrate is 5′-FAM labeled (*green circles*) and hybridizes with competitor ssDNA upon separation from the upper strand of the forked DNA substrate. The substrate DNA (12.5 nM) was incubated at 30 ˚C for 0 to 30 min in buffer containing competitor ssDNA (62.5 nM) and the indicated concentrations of *cc*DnaB-His (50 nM) and WT His-DciA or His-DciA variants (*A*). The products were separated using 6% PAGE. Representative gel images from the DciA-titration experiment (*B* and *F*) and the time-course experiment (*D*) are shown. Multiple independent reactions were conducted to generate reaction curves (*C* and *E*) and a bar graph (*G*). For *panel C*, the curves were generated by connecting the mean values of the data points. For *panel E*, the data were fitted using a three-parameter exponential model: a+b∗Exp(c∗x), where a, b, c and x represent asymptote, scale, growth rate, and the protein concentration, respectively. The fitted parameters are provided in Table S1. For *panel G*, statistical significance was assessed using Student's *t* test.

To elucidate the role of the DciA Arg106 and Leu119 residues in operation of the *cc*DnaB helicase, we performed an *in vitro* helicase assay using the forked DNA substrate. In this assay, the hexameric *cc*DnaB-His ring interacts with the 5′-end of ssDNA tail of the substrate and threads the 5′ ssDNA tail, followed by the subsequent migration of the ring in the 3′ direction with His-DciA, leading to the unwinding of the dsDNA region. The separated bottom strand DNA is then hybridized with a short 6-carboxyfluorescein (FAM)-labeled oligonucleotide present in the reaction mixture (18) (Fig. 6*A*). As previously reported, *cc*DnaB-His exhibited the substrate unwinding activity at a basal level (∼15%), which was markedly stimulated to ∼90% in the presence of His-DciA (Fig. 6, *B*–*E*). Notably, the His-DciA (R106A), His-DciA (L119S), and His-DciA (R106A L119S) variants retained their ability to promote the unwinding of the forked DNA substrate (Fig. 6, *B*–*G*), consistent with their ability to bind *cc*DnaB-His (Fig. 4). These results suggest that the DciA Arg106 and Leu119 residues are dispensable for interaction with the 5′ ssDNA tail and robust fork progression. In contrast, helicase activity was compromised in the presence of His-DciA (Δ1–45). Given that this variant largely retained the *cc*DnaB binding activity (Fig. 6, *F* and *G*), the results suggested that the N-terminal extension of DciA, unlike Arg106 and Leu119 residues within DUE-721, is important for stimulating fork progression.

### DciA (R106A) and DciA (L119S) impair topological loading of *cc*DnaB

**Figure 7:**
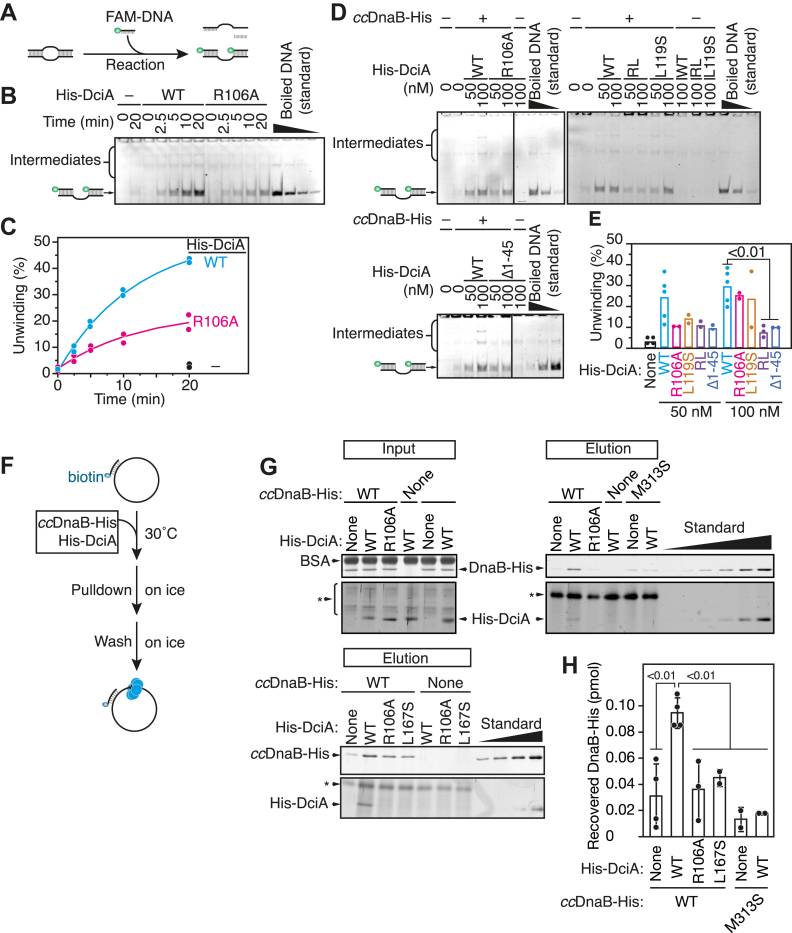
**DciA Arg106 and Leu119 are crucial for topological *cc*DnaB loading.***A*–*E*, topological loading assay using a bubble DNA substrate. Two-hundred nanomolars of WT His-DciA or His-DciA were incubated with *cc*DnaB-His (200 nM) and a bubble DNA substrate (12.5 nM) at 30 ˚C for 0 to 20 min in buffer containing 5′-FAM labeled competitor ssDNA (62.5 nM) (*B*, *C*). The curves were generated by using a three-parameter exponential model, as in Figure 6*E*. For the DciA-titration experiment, reactions were terminated after 20 min of incubation at 30 ˚C (*D*, *E*). Separation of the bubble DNA substrate allows the bottom strand to hybridize with competitor ssDNA. A schematic of the assay is shown in *panel A,* where *green circles* indicate the 5′-DNA ends labeled by FAM. The products were separated using 9% polyacrylamide gel electrophoresis. Representative gel images from the time-course experiment (*B*) and the DciA-titration experiment (*D*) are shown. Boiled DNA samples (5%, 10%, 25%, and 50% of input for *panel B*; 5, 25, 50% of input for *panel D*) were used for band intensity quantification and the values were plotted as unwinding (%) (*C*, *E*). For *panel C*, mean values at each time-point were used to generate reaction curves. For *panel E,* statistical significance was assessed using Student's *t* test. RL indicates R106A L119S double mutant. *F*–*H*, topological loading assay using a circular ssDNA substrate. Two hundred nanomolars of WT His-DciA or His-DciA (R106A) were incubated at 30 ˚C for 10 min in buffer containing *cc*DnaB-His (200 nM) and M13 ssDNA (8.4 nM) hybridized with biotinylated short ssDNA, followed by DNA pull-down using streptavidin magnetic beads. The beads were harvested, washed once in high-salt buffer (10 μl), and resuspended in sample buffer. The beads-bound materials were analyzed using SDS-12% polyacrylamide gel electrophoresis and silver staining. *cc*DnaB-His (M313S) and His-DciA (L167S) were used as negative controls. Ten percent of input samples were analyzed as loading control. Statistical significance was assessed using Student's *t* test. Standard samples were prepared by two-fold serial dilutions of 0.5 pmol proteins. The *asterisk* indicates an extra band derived from magnetic beads.

To elucidate the role of the DciA Arg106 and Leu119 residues in the topological loading of the *cc*DnaB helicase, we performed an *in vitro* helicase assay using a bubble-shaped DNA substrate comprising a 38-mer ssDNA region flanked by 21 bp duplex DNA regions (Fig. 7*A*). In this assay, loading of the hexameric DnaB structure, a closed ring as a default state, onto the ssDNA region within the substrate depends on topological isomerization of the structure which involves opening the ring to allow threading of the ssDNA region of the substrate. Subsequent migration toward the 3′ DNA end unwinds the dsDNA region and the separated bottom strand DNA hybridizes with a short FAM-labeled oligonucleotide in the reaction mixture (Fig. 7*A*). As reported previously, WT DnaB-His alone was virtually inactive in unwinding the substrate, but its activity was markedly enhanced in the presence of WT His-DciA (Fig. 7, *B* and *C*), demonstrating the critical role of DciA in helicase loading. In contrast, the His-DciA (R106A) and His-DciA (L119S) variants were less effective in stimulating the topological loading of *cc*DnaB-His, and this defect was further exacerbated by the double mutant His-DciA (R106A L119S) (Fig. 7, *B*–*E*). These results indicated that DciA Arg106 and Leu119 are crucial for the process of topological *cc*DnaB loading onto ssDNA.

To further validate this finding, we conducted a topological loading assessment using a pull-down assay with an M13 circular ssDNA substrate hybridized to a short biotinylated oligonucleotide. In this assay, the substrate was incubated with *cc*DnaB-His and the ssDNA-unloaded *cc*DnaB-His was washed out with a high-salt buffer. The remaining ssDNA-loaded *cc*DnaB-His was recovered using streptavidin magnetic beads (Fig. 7*F*). As expected, when the substrate was coincubated with *cc*DnaB-His and WT His-DciA, both proteins were readily pulled down (Fig. 7, *G* and *H*). This activity was diminished when either the DciA-binding-deficient *cc*DnaB-His (M313S) variant or the *cc*DnaB-binding-deficient His-DciA (L167S) variant, was used, supporting the validity of this assay. Notably, coincubation with His-DciA R106A significantly reduced the pull-down efficiency. The residual recovery of *cc*DnaB-His likely resulted from DciA-independent spontaneous loading, as similar basal levels of His-DnaB recovery were observed when *cc*DnaB-His was incubated alone or with the His-DciA (L167S) variant. These findings underscore the crucial role of DUF-721-mediated ssDNA binding in facilitating topological *cc*DnaB loading onto ssDNA.

Finally, we utilized His-DciA (Δ1–45) to examine the role of the N-terminal extension in topological helicase loading. Using an *in vitro* helicase assay using a bubble-shaped DNA substrate, we observed significant defects in the topological loading activity of this variant (Fig. 7, *D* and *E*). Together, these findings support the idea that ssDNA binding *via* the N-terminal extension of DUF-721 plays a specific role in both topological loading and fork progression.

## Discussion

Establishing replication forks requires a dedicated mechanism by which the ring-shaped replicative DNA helicase is loaded onto chromosome DNA, encircling single-stranded DNA (ssDNA). In most bacteria, the replicative helicase DnaB has coevolved with its partner DciA-family protein, which contains the conserved domain DUF-721. Despite its ubiquity, the biological and biochemical roles of DUF-721 in DnaB operation have remained unclear. Our study provides insights into this by identifying the critical contribution of the DciA Arg106 and Leu119 residues within DUF-721. Using a plasmid complementation assay, we found that Arg106 and Leu119 residues are essential for DciA functions *in vivo*. Biochemical assays further revealed that the DciA (R106A) and DciA (L119S) variants exhibit reduced affinity for ssDNA, which compromises the topological loading of *cc*DnaB onto ssDNA. However, these variants retain their ability to bind *cc*DnaB and promote helicase migration. This finding aligns with previous data showing that *V. cholerae* DciA variants impaired in ssDNA binding could still support the cognate DnaB helicase activity (24). Thus, our results support the idea that ssDNA binding of DciA *via* DUF-721 plays a specific and crucial role in topological helicase loading, distinct from its role in helicase migration once the DnaB ring is chromosomally loaded. To our knowledge, this study provides the first evidence of the biological significance of ssDNA binding to DciA DUF-721 *in vivo*.

This study also highlights the distinct role of the N-terminal extension of DUF-721 in DciA functions. Similar to the DciA (R106A L119S) double mutant, the DciA (Δ1–45) variant retains the ability to bind *cc*DnaB, but is defective in ssDNA binding and topological helicase loading. These findings support the idea that both DUF-721 and its N-terminal extension contribute to ssDNA binding, which collectively promotes topological helicase loading. Notably, the DciA (Δ1–45) variant also impairs translocation of the DNA-loaded *cc*DnaB, in contrast to the DciA (R106A L119S) double mutant, which maintains *cc*DnaB translocation activity. These observations suggest that ssDNA binding by the N-terminal extension of DUF-721 directly or indirectly contributes to *cc*DnaB dynamics during helicase translocation, while ssDNA binding by DUF-721 itself plays a specific role in topological helicase loading and is dispensable for helicase translocation.

**Figure 8:**
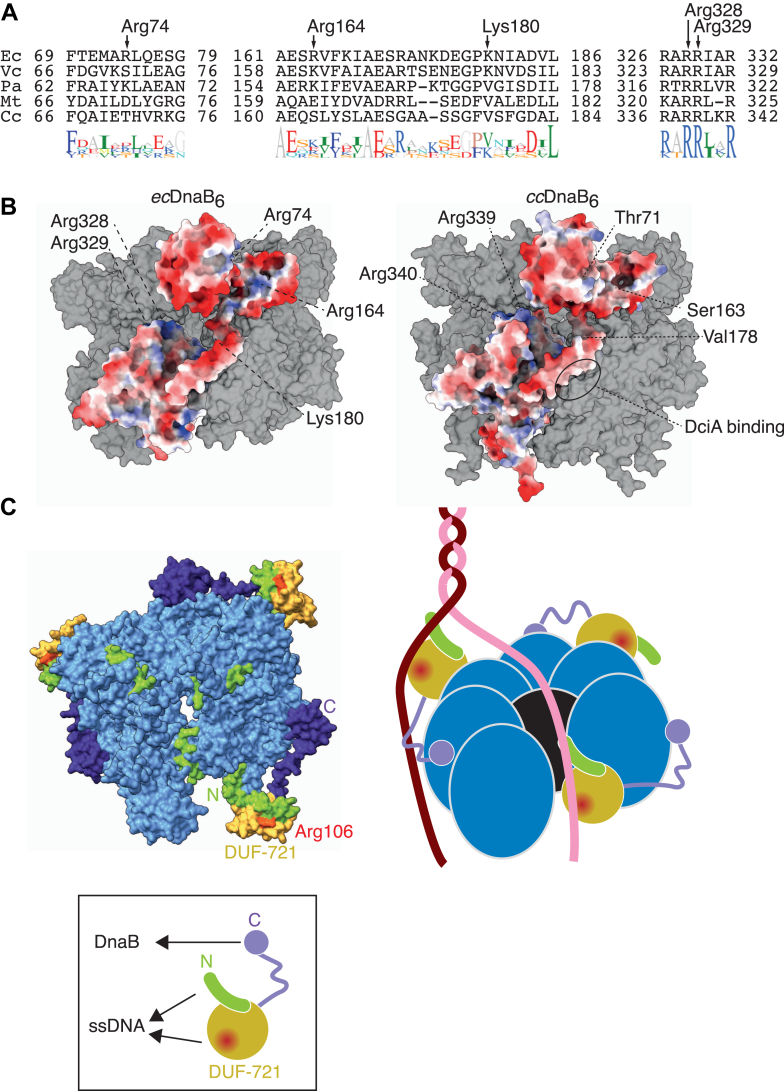
**A model for the *cc*DnaB-DciA loading system.***A*, multiple sequence alignment. The amino acid sequences of DnaB-family proteins from *V. choleare* (*Vc*), *P. aeruginosa* (*Pa*), *M. tuberculosis* (*Mt*), and *C. crescentus* (*Cc*), which are operated by the cognate DciA proteins, but not DnaC, are aligned with those of *ec*DnaB using Geneious Prime (https://www.geneious.com). The *ec*DnaB residues (Arg74, Arg164, Lys180, Arg328, and Arg329) involved in ssDNA binding at the exterior surface of the hexameric ring are indicated with *arrows*. *B*, structural comparison. The hexameric *ec*DnaB_6_ ring (*left*; PDBID: 6qem) and an AlphaFold-predicted hexameric *cc*DnaB_6_ ring (*right*) are shown in surface representation. Both rings are in the closed conformation. A single protomer is colored by surface electrostatic potential (*red*: negative potential; *white*: neutral; *blue*: positive potential), visualized using ChimeraX (44). For simplicity, the unstructured N-terminal region (1, 2, 3, 4, 5, 6, 7, 8, 9, 10, 11, 12, 13, 14, 15, 16, 17, 18, 19, 20, 21, 22, 23, 24, 25, 26, 36) of *cc*DnaB is omitted. The *ec*DnaB residues (Arg74, Arg164, Lys180, Arg328, and Arg329) and the counterpart *cc*DnaB residues are indicated. The *cc*DnaB residues (Val188, Phe305, Val308, and Met313) involved in DciA binding are highlighted as “DnaB binding.” *C*, an AlphaFold 3-based open structure model of the *cc*DnaB_6_ ring in complex with DciA (*left*) (45) and a schematic illustration of DnaB loading (*right*) are shown. A structure of a trimer comprising a single DciA molecule and two *cc*DnaB molecule was predicted using AlphaFold 3 and superimposed on the *Escherichia coli* DnaB-DnaC complex (PDBid: 6qel). For simplicity, only DciA (N-terminal extension in *green*, DUF-721 in *yellow*, and C-terminal extension in *purple*) and *cc*DnaB promoters (*blue*) are depicted. Arg106 is highlighted in *red*. Lagging and leading strands are colored in *dark red* and *pink*, respectively. Entry of the lagging strand into the gap of the open *cc*DnaB_6_ ring is promoted by DciA binding to the lagging strand and/or leading strand. This binding is mediated by DUF-721 and its N-terminal extension of DciA. While the schematic illustrates ssDNA binding of DUF-721 and its N-terminal extension occurring *in cis*, we do not exclude the possibility of *trans* binding: *e.g.* the leading and lagging strands may be recognized separately by DUF-721 and its N-terminal extension, respectively, within the same DciA protomer. DUF, domain of unknown function; PDB, Protein Data Bank.

How mechanistically does DciA stimulate *cc*DnaB loading? In *E. coli*, previous studies have shown that ssDNA binding to the exterior surface of the *ec*DnaB hexamer is important for helicase loading (27, 28). This interaction involves several basic residues (Arg74, Arg164, Lys180, Arg328, and Arg329) exposed on the hexamer's exterior surface (Fig. 8, *A* and *B*). Ala-substitutions of these residues disrupted or reduced ssDNA binding, with simultaneous Ala-substitution of Arg328 and Arg329 leading to inefficient *ec*DnaB loading onto ssDNA (27, 28). These findings suggest that ssDNA binding to the exterior surface of the *ec*DnaB hexamer plays a direct role during the loading process. While Arg328 and Arg329 are conserved across diverse species, the other basic residues crucial for ssDNA binding are less conserved in *cc*DnaB and the other DnaB-family proteins coevolved with the cognate DciA-family proteins (Fig. 8*A*). Moreover, electrostatic potential predictions indicate that the exterior surface of the *cc*DnaB hexamer is largely negatively-charged (Fig. 8*B*), which may hinder its interaction with ssDNA. We propose that the affinity of DciA for ssDNA overcomes this barrier, stimulating *cc*DnaB loading onto ssDNA. One possible mechanism is that DciA binds to the lagging strand to stimulate its passage into the central cavity of the *cc*DnaB ring (Fig. 8*C*). Alternatively, DciA may bind to the leading strand to accommodate the position of the gap in the open-spiral conformation of the *cc*DnaB ring in order to increase the accessibility of the opposite lagging strand (Fig. 8*C*). These two mechanisms are not mutually exclusive and could act in concert to enhance the efficiency of topological helicase loading. For instance, DUF-721 binds the leading and its N-terminal extension does the lagging strand, or the other way around. In either case, DciA Arg106 likely plays an important role in ssDNA interaction, with this activity being supported directly or indirectly by Leu119, thereby enhancing topological helicase loading. In this context, we infer that the flexible linker connecting between DUF-721 and the C-terminal DnaB binding helices may help dynamically position DUF-721 to optimize its interaction with ssDNA and helicase.

The mechanism of helicase loading system by which DciA interacts with ssDNA contrasts with the operation of the DnaC loader in *E. coli*. DnaC comprises the N-terminal *ec*DnaB binding domain and the C-terminal AAA+ domain responsible for ATP hydrolysis and ssDNA binding (6, 10). DnaC variants that lose the ssDNA binding activity fail to hydrolyze ATP and concomitantly show impaired DnaB loading (6). Structural studies suggest that the *ec*DnaB-DnaC complex undergoes a conformational change, opening the ring to engage ssDNA. Upon ssDNA binding, DnaC promotes ATP hydrolysis, which alters the conformational state of the *ec*DnaB-DnaC complex and presumably leads to DnaC dissociation from DNA-loaded *ec*DnaB (6, 29). The release of DnaC is stimulatory for installation of primase DnaG and is crucial for subsequent progression of the replication fork (30). Similar mechanism has been proposed in *B. subtilis*, where the AAA+ helicase loader DnaI, a counterpart of *E. coli* DnaC dissociates from the cognate replicative helicase upon ssDNA binding to activate helicase translocation (15). In contrast, our findings suggest that in the DciA system, ssDNA binding to DciA is required for efficient helicase loading, but unlike DnaC or DnaI, DciA remains associated with *cc*DnaB after loading. This interaction may play an active role in facilitating subsequent translocation of *cc*DnaB along the chromosome. Although we do not rule out the possibility that the cognate DnaG promotes DciA dissociation from *cc*DnaB based on an analogy in *E. coli* DnaG-DnaB-DnaC interaction (13), we speculate that DciA employs a distinct mechanism in maintaining helicase stability during the process of helicase loading and translocation, which may reflect broader evolutionary differences in how bacteria orchestrate helicase operation.

Our findings suggest that *E. coli* and *C. crescentus* DnaB proteins share the same contact surfaces for interaction with their cognate helicase loaders. This provides general insights into the conserved features of helicase-loader interactions. Structural analyses of the *ec*DnaB hexamer reveal that the hydrophobic α-helix within the RecA-fold domain, along with the linker helix of the adjacent protomer, forms the binding site for the N-terminal α-helix of DnaC. Biochemical studies further support the predominant role of hydrophobic interactions in the *ec*DnaB-DnaC interaction (10, 31). Importantly, the corresponding region in *cc*DnaB is also conserved in terms of hydrophobicity and is crucial for binding DciA. In line with this, hydrophobic leucine residues within the C-terminal α-helix of DciA are essential for its binding to ccDnaB (18). Thus, despite differences in the overall structures of DciA and DnaC, both share a similar mechanism for binding to DnaB through hydrophobic interactions. Consistent with this idea, we previously demonstrated that *C. crescentus* DciA can bind and stimulate the loading of *E. coli* DnaB (18). Similar orthogonal interactions have also been observed between *Vibrio* DciA and *ec*DnaB (25), further underscoring the conserved nature of this interaction.

Multiple DciA residues collectively contribute to form a contact surface that enhances overall ssDNA affinity. In *E. coli*, when *ec*DnaB accidently dissociates from the replication fork, the ssDNA-binding protein (SSB) bound to the lagging strand inhibits reloading of *ec*DnaB onto the stalled fork. To overcome this barrier, the DnaB-DnaC system requires remodeling of SSB-ssDNA interaction through actions of primosomal proteins including PriA, PriB, PriC, and DnaT (32, 33, 34, 35). Although PriA family proteins are ubiquitous across eubacteria, PriB, PriC, and DnaT are less conserved, suggesting that an alternative mechanism might facilitate remodeling of the SSB-ssDNA complex at stalled replication forks in other species. Given the importance of DciA-family proteins in replication restart (18, 22), we propose that the presence of multiple ssDNA binding residues within DciA might increase the accessibility of the DnaB-DciA complex to the SSB-coated lagging DNA, thereby facilitating helicase reloading under conditions where specialized remodeling proteins are absent or insufficient.

It should be noted that this extension exhibits substantial diversity in length and structure among DciA-family proteins (16). For example, the N-terminal extension of *C. crescentus* DciA is longer and contains more predicted helices than the corresponding regions of DciA homologs from *P. aeruginosa*, *V. cholerae*, and *Thermotoga maritima* (Fig. S4*A*). Given that the replicative helicase is a potential target of a phage-derived protein to modulate the host replication system to favor phage propagation (19, 36), host cells may benefit from robust helicase translocation mediated by the N-terminal extension of DUF-721. We speculate that differences in phage defense strategies among bacterial species may have driven the diversification of the N-terminal extension of DciA-family proteins.

## Experimental procedures

### Bacterial strains and proteins

The strains used in this study are listed in Table 1. *Caulobacter* strains were grown at 30 °C in PYE (2 g/L peptone, 1 g/L yeast extract, 8 mM magnesium sulfate, and 0.5 mM calcium chloride) medium supplemented with appropriate antibiotics, as described. When necessary, cumate (1 μM), glucose (0.1%), or xylose (0.1%) was added to the culture medium, as indicated. Recombinant DciA and *cc*DnaB proteins were purified as described previously (18). Bovine serum albumin (BSA) was purchased from Roche.

**Table 1:** Strains used in this study

Strain	Genotype	Reference
*Caulobacter crescentus*		
NA1000	wild-type *Caulobacter crescentus* strain	(46)
SHQ209	NA1000 *dciA*::P*xylX*-*dciA*	(18)
*Escherichia coli*		
DH5α	General cloning strain	Invitrogen
Rosetta 2 (DE3)	Strain for overexpression of recombinant protein	Novagen
BTH101	A Δ*cyaA* mutant strain for bacterial two-hybrid screening	(40)

### DNAs

The plasmids and oligonucleotides used in this study are listed in Tables 2 and 3, respectively.

**Table 2:** Plasmids used in this study

Plasmid	Description	Reference
pUT18	Derivative of the high copy number vector pUC19, including AmpR and, encoding the T18 fragment under the lac promoter. The T18 ORF lies upstream of MCS.	(40)
pKT25	Derivative of the low copy number vector pSU40, including kanR and, encoding the T25 fragment under the lac promoter. The T25 ORF lies downstream of MCS.	(40)
pUT18dciA	pUT18 plasmid containing T18-*dciA* fusion.	This study
pUT18dnaB	pUT18 plasmid containing T18-*dnaB* fusion.	This study
pKT25dnaB	pKT25 plasmid containing T25-*dnaB* fusion.	This study
pKT25dnaB (1–158)	pKT25 plasmid containing T25-*dnaB* (1–158) fusion	This study
pKT25dnaB (1–202)	pKT25 plasmid containing T25-*dnaB* (1–202) fusion.	This study
pKT25dnaB (159–507)	pKT25 plasmid containing T25-*dnaB* (159–507) fusion.	This study
pKT25dnaB (203–507)	pKT25 plasmid containing T25-*dnaB* (203–507) fusion.	This study
pKT25dnaB (148–507)	pKT25 plasmid containing T25-*dnaB* (148–507) fusion.	This study
pKT25dnaB (1–127)	pKT25 plasmid containing T25-*dnaB* (1–127) fusion.	This study
pKT25dnaB (D182A)	pKT25 plasmid congaing T25-*dnaB (D182A)* fusion.	This study
pKT25dnaB (L184S)	pKT25 plasmid congaing T25-*dnaB (L184A)* fusion.	This study
pKT25dnaB (V188S)	pKT25 plasmid congaing T25-*dnaB (V188S)* fusion.	This study
pKT25dnaB (E189A)	pKT25 plasmid congaing T25-*dnaB(E189A)* fusion.	This study
pKT25dnaB (E193A)	pKT25 plasmid congaing T25-*dnaB (E193A)* fusion.	This study
pKT25dnaB (F305S)	pKT25 plasmid congaing T25-*dnaB (F305A)* fusion.	This study
pKT25dnaB (V308S)	pKT25 plasmid congaing T25-*dnaB (V308A)* fusion.	This study
pKT25dnaB (D310A)	pKT25 plasmid congaing T25-*dnaB (D310A)* fusion.	This study
pKT25dnaB (M313S)	pKT25 plasmid congaing T25-*dnaB (M313S)* fusion.	This study
pKT25dnaB (E314A)	pKT25 plasmid congaing T25-*dnaB(E314A)* fusion.	This study
pET28a	A kanamycin-resistant pET expression vector	Novagen
pET28aCCNA01737cHis	A pET28a derivative expressing CCNA_01737 (DnaB) with a C-terminal His6 tag	(18)
pET28aDnaBcHis (V188S)	A pET28a derivative expressing DnaB (L188S) with a C-terminal His6 tag	This study
pET28aDnaBcHis (F305S)	A pET28a derivative expressing DnaB (V305S) with a C-terminal His6 tag	This study
pET28aDnaBcHis (V308S)	A pET28a derivative expressing DnaB (F308S) with a C-terminal His6 tag	This study
pET28aDnaBcHis (M313S)	A pET28a derivative expressing DnaB (M313S) with a C-terminal His6 tag	This study
pMR10	A kanamycin-resistant RK2-based low copy plasmid	(47)
pMR10dciA	A pMR10 derivative expressing wild-type DciA	(18)
pMR10dciA (F45A)	A pMR10 derivative with the *dciA (F45A)* allele	This study
pMR10dciA (E58A)	A pMR10 derivative with the *dciA (E58A)* allele	This study
pMR10dciA (V60A)	A pMR10 derivative with the *dciA (V60A)* allele	This study
pMR10dciA (D62A)	A pMR10 derivative with the *dciA (D62A)* allele	This study
pMR10dciA (T63A)	A pMR10 derivative with the *dciA (T63A)* allele	This study
pMR10dciA (L64S)	A pMR10 derivative with the *dciA (L64S)* allele	This study
pMR10dciA (R66A)	A pMR10 derivative with the *dciA (R66A)* allele	This study
pMR10dciA (R67A)	A pMR10 derivative with the *dciA (R67A)* allele	This study
pMR10dciA (P70A)	A pMR10 derivative with the *dciA(P70A)* allele	This study
pMR10dciA (R72A)	A pMR10 derivative with the *dciA (R72A)* allele	This study
pMR10dciA (K75A)	A pMR10 derivative with the *dciA (K75A)* allele	This study
pMR10dciA (R77A)	A pMR10 derivative with the *dciA (R77A)* allele	This study
pMR10dciA (L86S)	A pMR10 derivative with the *dciA (L86S)* allele	This study
pMR10dciA (R87A)	A pMR10 derivative with the *dciA (R87A)* allele	This study
pMR10dciA (Q99A)	A pMR10 derivative with the *dciA (Q99A)* allele	This study
pMR10dciA (Q102A)	A pMR10 derivative with the *dciA (Q102A)* allele	This study
pMR10dciA (R106A)	A pMR10 derivative with the *dciA (R106A)* allele	This study
pMR10dciA (L107S)	A pMR10 derivative with the *dciA (L107S)* allele	This study
pMR10dciA (M109S)	A pMR10 derivative with the *dciA (M109S)* allele	This study
pMR10dciA (L110S)	A pMR10 derivative with the *dciA (L110S)* allele	This study
pMR10dciA (L111S)	A pMR10 derivative with the *dciA (L111S)* allele	This study
pMR10dciA (K113A)	A pMR10 derivative with the *dciA (K113A)* allele	This study
pMR10dciA (V116A)	A pMR10 derivative with the *dciA (V116A)* allele	This study
pMR10dciA (R118A)	A pMR10 derivative with the *dciA (R118A)* allele	This study
pMR10dciA (L119S)	A pMR10 derivative with the *dciA (L119S)* allele	This study
pMR10dciA (3L)	A pMR10 derivative with the *dciA (L163S, L167S, L170S)* alleles	This study
pBXMCS2sfTq2	pBXMCS-2 derivative with sfTq2	(37)
pETsfTq2dciA (WT)-d	A pET28a derivative expressing sfTq2-DciA	(18)
pETsfTq2dciA(R106A)-d	A pETsfTq2dciA (WT)-d derivative with the *dciA(R106A)* allele	This study
pETsfTq2dciA(3L)-d	A pETsfTq2dciA (WT)-d derivative with the *dciA(L163S L167S L170S)* allele	This study
pET21a	An ampicillin-resistant pET expression vector	Novagen
pET21aHisdciA	A pET21a derivative with His-DciA	(18)
pET21aHisdciA (R106A)	A pET21aHisdciA derivative with the *dciA (R106A)* allele	This study
pET21aHisdciA (L167S)	A pET21aHisdciA derivative with the *dciA (L167S)* allele	(18)
pET21aHisdciA (L119S)	A pET21aHisdciA derivative with the *dciA (L119S)* allele	This study
pET21aHisdciA (R106AL119S)	A pET21aHisdciA derivative with the *dciA (R106AL119S)* allele	This study
pET21aHisdciA (4A)	A pET21aHisdciA derivative with the *dciA(*H98A Q99A Q102A R106A) allele	This study
pET21aHisdciA (4A2S)	A pET21aHisdciA derivative with the *dciA*(H98A Q99A Q102A R106A M109S L110S) allele	This study
pET21aHisdciA (Δ1–45)	A pET21aHisdciA derivative expressing a variant lacking DciA residues 1–45.	This study
pLacQFlacImCherry	A plasmid bearing the LacI-mCherry-coding region.	(38)
pMR10-mChdciA	A pMR10 vector bearing *dciA* with its native promoter.	This study
pMR10-mChdciA (L86S)	A pMR10-mChdciA derivative with a *dciA* allele (*L86S*)	This study
pMR10-mChdciA (R106A)	A pMR10-mChdciA derivative with a *dciA* allele (*R106A*)	This study
pMR10-mChdciA (L119S)	A pMR10-mChdciA derivative with a *dciA* allele (*L119S*)	This study

**Table 3:** Oligonucleotides used in this study

Name	Sequence (5′-)
291	GCCAAGCTTGTCAATGGTCCGATCCCGTCTC
294	ACCGAATTCGGTGCCCGGAAAGACGCCGCCG
307	GGGACTAGTCGCCGGGGCGATCGTCGGTGAC
308	TGTTATCCTCCTCGCCCTTGCTCACCATGCGAGGAAACTAGCATGGGGCC
309	GGCCCCATGCTAGTTTCCTCGCATGGTGAGCAAGGGCGAGGAGGATAACA
310	CTTCCGGCGTGGGCAGGGGGCGACGGGATCCACCGCCTCCCTTGTACAGCTCGTCCATGCCGCCG
606	ATCAAGCTTCTAGCGCTCCGAAGACAGGACCCCG
607	TAACATATGCCGATGTCTCTCGTCCCTGCGCT
645	AGTGCGGCCGCCTCGTCCGACGGAATATTCCGCGCC
808	CCCTCTAGAAATAATTTTGTTTAACTTTAAGAAGGAGATATACCATGGGCAGCAGCCATCACCATCATCATxCACCGTCGCCCCCTGCCCACGCCGGAAG
864	TTTTTTTTTTTTTTTTTTTTTTTTTTTTTTTGCCCTGTGGATAACAAGGATCCGGCTTTTAAGATCAACAACCTGGAAA
871	TTTCCAGGTTGTTGATCTTAAAAGCCGGATCCTTGTTATCCACAGGGCTTTTTTTTTTTTTTTTTTTTTTTTTTTTTTT
872	TTTCCAGGTTGTTGATCTTAAAAGCCGGATCCTTGTTATCCACAGGGC
894	TCGGAGAACTATATCGCACAATTTTTTTTTTTTTTTTTTTTTTTTTTTTTTTTTTTTTTCTGCCCTGTGGATAACAAGGA
895	TCCTTGTTATCCACAGGGCAGTTTTTTTTTTTTTTTTTTTTTTTTTTTTTTTTTTTTTTTTGTGCGATATAGTTCTCCGA
896	TCGGAGAACTATATCGCACAA
897	CTGCCCTGTGGATAACAAGGA
1119	GGAGATCGTCGGCGACACCCTGGCGGCGCGCACCGAGCCGGTCCGCATCATC
1120	GATGATGCGGACCGGCTCGGTGCGCGCCGCCAGGGTGTCGCCGACGATCTCC
1121	GATCGTCGGCGACACCCTGGCGCGGGCCACCGAGCCGGTCCGCATCATCAAG
1122	CTTGATGATGCGGACCGGCTCGGTGGCCCGCGCCAGGGTGTCGCCGACGATC
1152	GCGCACCGAGCCGGTCCGCATCATCGCGGGCCGCAACGGCGAAGGCGGGGCC
1153	GGCCCCGCCTTCGCCGTTGCGGCCCGCGATGATGCGGACCGGCTCGGTGCGC
1154	CGAGCCGGTCCGCATCATCAAGGGCGCCAACGGCGAAGGCGGGGCCCTGGAG
1155	CTCCAGGGCCCCGCCTTCGCCGTTGGCGCCCTTGATGATGCGGACCGGCTCG
1156	CGCCAGGCTCGATATGCTGCTGGGCGCGGGCGTGGTCACCCGCCTGCGGATC
1157	GATCCGCAGGCGGGTGACCACGCCCGCGCCCAGCAGCATATCGAGCCTGGCG
1169	GTAGGATCCCGTCGCCCCCTGCCCACGCCGGAA
1204	CTGCAGGCCCGCTGGAAGGAGATCGCCGGCGACACCCTGGCGCGGCGCACC
1205	GGTGCGCCGCGCCAGGGTGTCGCCGGCGATCTCCTTCCAGCGGGCCTGCAG
1206	CGACACCCTGGCGCGGCGCACCGAGGCGGTCCGCATCATCAAGGGCCGCAA
1207	TTGCGGCCCTTGATGATGCGGACCGCCTCGGTGCGCCGCGCCAGGGTGTCG
1222	AACGGCGAAGGCGGGGCCCTGGAGTCGCGGGTCGACGGGCCGGTCGCGTCG
1223	CGACGCGACCGGCCCGTCGACCCGCGACTCCAGGGCCCCGCCTTCGCCGTT
1224	ACATCAGGCGCCGCAGATCACCGCCGCGCTCGATATGCTGCTGGGCAAGGGC
1225	GCCCTTGCCCAGCAGCATATCGAGCGCGGCGGTGATCTGCGGCGCCTGATGT
1226	GATATGCTGCTGGGCAAGGGCGTGGCCACCCGCCTGCGGATCGTGCAGGGC
1227	GCCCTGCACGATCCGCAGGCGGGTGGCCACGCCCTTGCCCAGCAGCATATC
1228	GCTGCTGGGCAAGGGCGTGGTCACCGCCCTGCGGATCGTGCAGGGCCCGGTC
1229	GACCGGGCCCTGCACGATCCGCAGGGCGGTGACCACGCCCTTGCCCAGCAGC
1230	GCTGGGCAAGGGCGTGGTCACCCGCTCGCGGATCGTGCAGGGCCCGGTCAAG
1231	CTTGACCGGGCCCTGCACGATCCGCGAGCGGGTGACCACGCCCTTGCCCAGC
1237	GCGGGATCCCGTGCCGATGTCTCTCGTCCCTGCGC
1238	GGGGGTACCTCGTCCGACGGAATATTCCGCGCC
1239	TCCGGATCCCCGTCGCCCCCTGCCCACGCCGGAAG
1240	CCCGGTACCCCGCGCTCCGAAGACAGGACCCCGCGA
1241	CCGGGTACCTAAGTAACTAAGAATTCGGCCGTCG
1242	GTCGGATCCGCAGCCCGCCGCGTGCGCGCCAGGT
1244	CTGGGATCCTGCAAGCTTGGCGTAATCATGGTCA
1245	CTCGGTACCAGCCGCCAGCGAGGCCACGGGCGG
1436	CAAGCTGCTAGCGCTCCGAAGACAGGACCCCG
1453	ATCAAGCTTCTAGCGCTCCGAAGACAGGACCCCGCGACCCGACTTCAGCGAGGCCTGTTTGGACGCGCCGTCGGGC
1456	TGTTCCGGTACCTCGATCTGATCGCGGGCGTTACG
1457	GTCGAGGGTACCGACATGCCGCCGTCGCGGCTATA
1458	CGGCGGGGATCCGGGCGTCTCGACCGACCTGATGGACC
1459	CGATCAGGATCCGGCGGCGGAACAGAGCCTCTACAGCC
1514	CCTGCTGAAGGACCTGGAAGACCGCGCCGGCAAGGGTCCCGCCGCGCTGCAG
1515	CTGCAGCGCGGCGGGACCCTTGCCGGCGCGGTCTTCCAGGTCCTTCAGCAGG
1516	GCCGCGCTGCAGGCCCGCTGGAAGGCGATCGTCGGCGACACCCTGGCGCGG
1517	CCGCGCCAGGGTGTCGCCGACGATCGCCTTCCAGCGGGCCTGCAGCGCGGC
1518	CCTGGCGCGGCGCACCGAGCCGGTCGCCATCATCAAGGGCCGCAACGGCGAA
1519	TTCGCCGTTGCGGCCCTTGATGATGGCGACCGGCTCGGTGCGCCGCGCCAGG
1522	CGGCGAAGGCGGGGCCCTGGAGTTGGCGGTCGACGGGCCGGTCGCGTCGCTG
1523	CAGCGACGCGACCGGCCCGTCGACCGCCAACTCCAGGGCCCCGCCTTCGCCG
1526	TCAGGCGCCGCAGATCACCGCCAGGTCGGATATGCTGCTGGGCAAGGGCGTGG
1527	CCACGCCCTTGCCCAGCAGCATATCCGACCTGGCGGTGATCTGCGGCGCCTGA
1528	GATCACCGCCAGGCTCGATATGCTGTCGGGCAAGGGCGTGGTCACCCGCCTG
1529	CAGGCGGGTGACCACGCCCTTGCCCGACAGCATATCGAGCCTGGCGGTGATC
1551	TAGGCGGGTACCTTGGCGATGTTGAAGGCGATGTTG
1598	GGCCCGCTGGAAGGAGATCGTCGGCGCCACCCTGGCGCGGCGCACCGAGCCGG
1599	CCGGCTCGGTGCGCCGCGCCAGGGTGGCGCCGACGATCTCCTTCCAGCGGGCC
1600	CCGCTGGAAGGAGATCGTCGGCGACGCCCTGGCGCGGCGCACCGAGCCGGTCC
1601	GGACCGGCTCGGTGCGCCGCGCCAGGGCGTCGCCGACGATCTCCTTCCAGCGG
1602	CTGGAAGGAGATCGTCGGCGACACCTCGGCGCGGCGCACCGAGCCGGTCCGCA
1603	TGCGGACCGGCTCGGTGCGCCGCGCCGAGGTGTCGCCGACGATCTCCTTCCAG
1606	GCCGGTCGCGTCGCTGATCCAACATGCCGCGCCGCAGATCACCGCCAGGCTCG
1607	CGAGCCTGGCGGTGATCTGCGGCGCCGGATGTTGGATCAGCGACGCGACCGGC
1608	GTCGCTGATCCAACATCAGGCGCCGGCCATCACCGCCAGGCTCGATATGCTGC
1609	GCAGCATATCGAGCCTGGCGGTGATCGGCGGCGCCTGATGTTGGATCAGCGAC
1614	GCCGCAGATCACCGCCAGGCTCGATTCGCTGCTGGGCAAGGGCGTGGTCACCC
1615	GGGTGACCACGCCCTTGCCCAGCAGCGAATCGAGCCTGGCGGTGATCTGCGGC
1616	GCAGATCACCGCCAGGCTCGATATGTCGCTGGGCAAGGGCGTGGTCACCCGCC
1617	GGCGGGTGACCACGCCCTTGCCCAGCGACATATCGAGCCTGGCGGTGATCTGC
1655	AGGGGATCCCGACGAAAAGCGTAACGCCCGCGATC
1710	GCCTCTTCGGGCTTCGTGTCCTTCGGCGCCGCCCTGCGCGGCGCGGTCGAGATGACC
1711	GGTCATCTCGACCGCGCCGCGCAGGGCGGCGCCGAAGGACACGAAGCCCGAAGAGGC
1712	TCGGGCTTCGTGTCCTTCGGCGACGCCTCGCGCGGCGCGGTCGAGATGACCGCCGAG
1713	CTCGGCGGTCATCTCGACCGCGCCGCGCGAGGCGTCGCCGAAGGACACGAAGCCCGA
1714	TCCTTCGGCGACGCCCTGCGCGGCGCGTCCGAGATGACCGCCGAGGCCTATAGCCGC
1715	GCGGCTATAGGCCTCGGCGGTCATCTCGGACGCGCCGCGCAGGGCGTCGCCGAAGGA
1716	TTCGGCGACGCCCTGCGCGGCGCGGTCGCGATGACCGCCGAGGCCTATAGCCGCGAC
1717	GTCGCGGCTATAGGCCTCGGCGGTCATCGCGACCGCGCCGCGCAGGGCGTCGCCGAA
1718	CTGCGCGGCGCGGTCGAGATGACCGCCGCGGCCTATAGCCGCGACGGCGGCATGTCG
1719	CGACATGCCGCCGTCGCGGCTATAGGCCGCGGCGGTCATCTCGACCGCGCCGCGCAG
1720	CGTAAGGGCGAGATCGACGCCAGCGAGAGCGGCCGCGTGCGCGACGCGGCCATGGAG
1721	CTCCATGGCCGCGTCGCGCACGCGGCCGCTCTCGCTGGCGTCGATCTCGCCCTTACG
1722	GAGATCGACGCCAGCGAGTTCGGCCGCTCGCGCGACGCGGCCATGGAGCTGCAGAAC
1723	GTTCTGCAGCTCCATGGCCGCGTCGCGCGAGCGGCCGAACTCGCTGGCGTCGATCTC
1724	GACGCCAGCGAGTTCGGCCGCGTGCGCGCCGCGGCCATGGAGCTGCAGAACGCGCCG
1725	CGGCGCGTTCTGCAGCTCCATGGCCGCGGCGCGCACGCGGCCGAACTCGCTGGCGTC
1726	GAGTTCGGCCGCGTGCGCGACGCGGCCAGCGAGCTGCAGAACGCGCCGCTCTATATC
1727	GATATAGAGCGGCGCGTTCTGCAGCTCGCTGGCCGCGTCGCGCACGCGGCCGAACTC
1728	TTCGGCCGCGTGCGCGACGCGGCCATGGCGCTGCAGAACGCGCCGCTCTATATCGAC
1729	GTCGATATAGAGCGGCGCGTTCTGCAGCGCCATGGCCGCGTCGCGCACGCGGCCGAA
1758	[BIOTIN]-GGGTAACGCCAGGGTTTTCCCAGTCACGAC
1790	[FAM]-TCCTTGTTATCCACAGGGCAGTTTTTTTTTTTTTTTTTTTTTTTTTTTTTTTTTTTTTTTTGTGCGATATAGTTCTCCGA
2201	[FAM]-TTTTTTTTTTTTTTTTTTTTTTTTTTTTTTTTTTT
2341	CGGGTCGACGGGCCGGTCGCGTCGCTGATCCAAGCTGCGGCGCCGCGCATCACCGCCGCGCTCGATATGCTGC
2342	CGGGTCGACGGGCCGGTCGCGTCGCTGATCCAAgcTgcGGCGCCGCgcATCACCGCCGCGCTCGATtcGtcGCTGGGCAAGGGCGTGGTC
2390	GGGGgatCcGTGATGATGATGGTGATGGCTGCTGCCCATGGTATATCTCCTTC
2393	GACgGaTcCGGCAAGGGTCCCGCCGCGCTGCAGG

To construct pUT18dciA and pUT18dnaB, the insert DNA fragments were amplified by PCR using NA1000 genomic DNA and primers (1239/1240 for *dciA* (0.56 kb); 1237/1238 for *ccdnaB* (1.5 kb)). A 3.0-kb vector DNA fragment was amplified by PCR using pUT18 and primers 1244/1245. After digestion with BamHI and KpnI, the insert and vector DNA fragments were ligated.

To construct pKT25dnaB, a 1.5 kb insert DNA fragment was amplified by PCR using NA1000 genomic DNA and primers 1237/1238. A 3.4-kb vector DNA fragment was amplified by PCR using pKT25 and primers 1241/1242. After digestion with BamHI and KpnI, the insert and vector DNA fragments were ligated. To construct pKT25dnaB (1–158) and pKT25dnaB (1–202), pKT25dnaB (1–247), the DNA fragments were amplified by PCR using pKT25dnaB and primers (1241/1456 for pKT25dnaB (1–158), 1241/1457 for pKT25dnaB (1–202), 1241/1551 for pKT25dnaB (1–247)). The products were digested with KpnI, followed by self-ligation.

To construct pKT25dnaB (159–507), pKT25dnaB (203–507), and pKT25dnaB (148–507), the DNA fragments were amplified by PCR using pKT25dnaB and primers (1242/1459 for pKT25dnaB (159–507), 1242/1458 for pKT25dnaB (203–507), and 1242/1655 for pKT25dnaB (148–507)). The products were digested with BamHI, followed by self-ligation.

To construct pKT25dnaB derivatives with a *ccdnaB* allele, the 1.5 kb insert DNA fragments were generated by overlap extension PCR using NA1000, primers 1237/1238, and mutagenic primers (1710/1711 for D182A 1712/1713 for L184S, 1714/1715 for V188S, 1716/1717 for E189A, 1718/1719 for E193A, 1720/1721 for F305S, 1722/1723 for V308S, 1724/1725 for D310A, 1726/1727 for M313S, and 1728/1729 for E314A). After digestion with BamHI and KpnI, the products were ligated to the BamHI-KpnI fragment of pKT25dnaB.

pET28aCCNA01737cHis is an overproducer for CcDnaB with a C-terminal hexahistidine tag (18). To construct its derivatives with a *ccdnaB* allele (V188S, F305S, V308S, or M313S), a 1.5 kb insert DNA fragment was amplified by PCR using primers 607/645, and template DNA pKT25dnaB (V188S), pKT25dnaB (F305S), pKT25dnaB (V308S), or pKT25dnaB (M313S). After digestion with NdeI and NotI, the products were ligated to the NdeI-NotI fragment of the vector pET28a.

pMR10dciA is a kanamycin-resistant low-copy number plasmid bearing *dciA* (18). To construct its derivatives with a *dciA* allele, the 1.0 kb insert DNA fragments were generated by overlap extension PCR using NA1000, primers 291/294, and mutagenic primers (1514/1515 for F45A, 1516/1517 for E58A, 1204/1205 for V60A, 1598/1599 for D62A, 1600/1601 for T63A, 1602/1603 for L64S, 1119/1120 for R66A, 1121/1122 for R67A, 1206/1207 for P70A, 1518/1519 for R72A, 1152/1153 for K75A, 1154/1155 for R77A, 1222/1223 for L86S, 1522/1523 for R87A, 1606/1607 for Q99A, 1608/1609 for Q102A, 1225/1226 for R106A, 1526/1527 for L107S, 1614/1615 for M109S, 1616/1617 for L110S, 1528/1529 for L111S, 1156/1157 for K113A, 1226/1227 for V116A, 1228/1229 for R118A, 1230/1231 for L119S). After digestion with EcoRI and HindIII, the products were ligated to the EcoRI-HindIII fragment of the vector pMR10.

pETsfTq2dciA (WT)-d is an overproducer for the N terminally superfolder mTurquoise2-tagged DciA (18, 37, 38). To construct its derivatives with a *dciA* allele, the 0.54 kb insert DNA fragments were amplified by PCR using primers 1169/1436 and templated DNA (pMR10dciA (R106A) for pET28-sfTq2dciA (R106A), pMR10dciA (3L) for pET28-sfTq2dciA (3L)). After digestion with BamHI and NheI, the products were ligated to the BamHI-NheI fragment of pETsfTq2dciA (WT)-d. pMR10dciA (3L) was generated by cloning a 1.0 kb PCR fragment using NA1000 and primers 294/1453 on pMR10.

To construct pET21aHisdciA (R106A), a 0.6 kb DNA fragment was amplified by PCR using pMR10dciA (R106A) and primers 808/606. After digestion with XbaI and HindIII, the products were ligated to the XbaI-HindIII fragment of pET21a. pET21aHisdciA (L119S) was generated similarly using pMR10dciA (L119S), instead of pMR10dciA (R106A).

To construct pET21aHisdciA (R106AL119S), a 0.6 kb DNA fragment was generated by overlap extension PCR using pMR10dciA (R106A) and primers 808/1231/1230/606. After digestion with NcoI and HindIII, the products were ligated to the NcoI-HindIII fragment of pET21aHisdciA (R106A).

To construct pET21aHisdciA (4A), a 0.3 kb DNA fragment was amplified by PCR using pET21aHisdciA (R106A) and primers 2341/606. After digestion with SalI and HindIII, the products were ligated to the SalI-HindIII fragment of pET21aHisdciA (R106A). pET21aHisdciA (4A2S) was generated similarly using primers 2342, instead of 2341.

To construct pET21aHisdciA (Δ1–45) a 5.9 kb DNA fragment was amplified by PCR using pET21aHisdciA and primers 2390/2393. After digestion with BamHI, the products were self-ligated.

To construct pMR10-mChdciA, a 1.3-kb DNA fragment consisting of the *dciA* promoter and mCherry-coding region was amplified by overlap extension PCR (NA1000 and primers 307/308; pLacQFlacImCherry and primers 309/310). This fragment and primers 294/310 were used to PCR amplify a 1.2-kb DNA fragment. Next, a 0.59 kb DNA fragment containing the DciA-coding region was generated by EcoRI and BamHI digestion of a 1.0-kb DNA fragment amplified by PCR using pNPTS3FdciA (18) and primers 291/294. These two DNA fragments were ligated to the EcoRI-BamHI fragment of pMR10. To construct pMR10-mChdciA derivatives with a *dciA* allele, mutagenic primers were used (1222/1223 for L86S, 1224/1225 for R106A, 1230/1231 for L119S) to generate a 0.59 kb DNA fragment.

### Bacterial two hybrid assay

This assay was performed as described (39, 40). Briefly, BTH101 cells were transformed by cointroducing pUT18dciA and pKT25-dnaB derivatives. Colonies were grown at 30 ˚C on LB agar supplemented with kanamycin (25 μg/ml), ampicillin (100 μg/ml), and 5-bromo-4-chloro-3-indolyl-β-D-galactopyranoside (X-Gal; 40 μg/ml), M63 agar supplemented with kanamycin (50 μg/ml), ampicillin (100 μg/ml), maltose (0.2%) and X-Gal (40 μg/ml), or MacConkey agar supplemented with kanamycin (50 μg/ml) and ampicillin (100 μg/ml), and maltose (0.2%).

### Size-exclusion chromatography

This assay was performed as described (18, 31, 37, 41). Briefly, proteins were loaded onto a Superdex 200 PC3.2/30 column (2.4-ml column volume), equilibrated with SEC buffer (25 mM Tris–HCl [pH 7.5], 300 mM sodium chloride, 20% glycerol, 0.1 mM ATP, and 5 mM magnesium chloride), and fractionated at a flow rate of 0.02 ml/min.

### ATPase assay

This assay was performed as described (18, 42, 43). Briefly, DnaB-His (50 nM) and His-DciA (100 nM) were incubated in buffer (20 mM Tris–HCl [pH 7.5], 10 mM magnesium acetate, 50 mM potassium glutamate, 0.1 mg/ml BSA) containing various concentrations of ATP for 5 min at 30 ˚C. Reactions were terminated by addition of a solution (10 mM Tris–HCl [pH 8.0], 1 mM EDTA, 0.1% SDS, 1 mM ATP, and 1 mM ADP), and a portion (0.5 μl) was spotted on a TLC PEI Cellulose plate (Merck). The plate was developed using a 1 M HCOOH/0.5 M LiCl solution.

### Electrophoretic mobility shift assay

His-DciA and 12.5 nM of the 5′-FAM-labeled oligonucleotide (2201 for 35-mer and 1790 for 80-mer) were incubated on ice for 5 min in buffer (10 μl; 20 mM Tris–HCl [pH 7.5], 10 mM magnesium acetate, 50 mM or 150 mM potassium glutamate, 0.1 mg/ml bovine serum albumin, 10% glycerol, and 2 mM ATP). A portion (9 μl) was analyzed by electrophoresis on 6% polyacrylamide gel.

### *In vitro* assays for helicase activity

The *in vitro* unwinding assay using a forked DNA substrate was performed essentially as described previously (18, 19, 31). The DNA substrate comprised 48 bp duplex DNA flanking 31-mer single-stranded tails. DnaB-His, His-DciA, and the DNA substrate (12.5 nM) were incubated at 30 ˚C in buffer (20 mM Tris–HCl [pH 7.5], 10 mM magnesium acetate, 50 mM potassium glutamate, 0.1 mg/ml BSA, and 2 mM ATP) containing 48-mer synthetic oligonucleotide 872 (62.5 nM) as a competitor. After reactions were terminated by addition of phenol/chloroform/isoamyl alcohol solution, DNA samples were extracted and analyzed using 6% PAGE. The substrate was generated by annealing synthetic DNA strands 864 and fluorescently-labeled 871.

For the helicase loading assay using a replication bubble-mimetic DNA substrate comprising a ssDNA bubble intervened by 21 bp duplex DNA regions (18), the annealed product of synthetic oligonucleotides (894/895 for 38-mer ssDNA bubble; 1400/1401 for 28-mer ssDNA bubble; 1402/1403 for 18-mer ssDNA bubble) was used instead of a forked DNA substrate, and competitor DNA was replaced by a mixture of fluorescently-labeled 21-mer oligonucleotides (896 and 897; 31.3 nM each). DNA samples were extracted and analyzed using 9% PAGE.

For the helicase loading assay using M13 DNA, proteins were incubated in pull-down buffer (10 μl; 20 mM Tris–HCl pH 7.5, 10 mM magnesium acetate, 50 mM potassium glutamate, 0.1 mM ATP, 0.1 mg/ml BSA) for 10 min at 30 ˚C. After addition of the streptavidin magnetic beads (5 μl slurry), samples were incubated on ice for 10 min. The beads were collected, washed once in pull-down buffer (10 μl) devoid of BSA, and resuspended in sample buffer. Following SDS-12% PAGE, proteins retained on the beads were detected using silver staining.

### Western blotting

The Anti-mCherry antibody purchased from GeneTex was used as primary antibody, which was detected by alkaline phosphatase-conjugated anti-mouse second antibodies (Bio-Rad). The immune reactions were performed in Immunoreaction Enhancer Solution (Toyobo) according to the manufacturer's instructions.

## Data availability

All data are available in the article.

## Supporting information

This article contains supporting information.

## Conflict of interest

The authors declare that they have no conflicts of interest with the contents of this article.
