# Supplementary material for: DUF-721 and N-terminal extension of the helicase loader DciA bind ssDNA to promote replicative DnaB helicase loading in Caulobacter crescentus
Source: J Biol Chem. 2025 Sep 15;301(10):110724. doi: 10.1016/j.jbc.2025.110724 (PMC12547741; doi:10.1016/j.jbc.2025.110724)
Supplement: Figures S1–S4 [file mmc3.docx]

**Supporting Information**


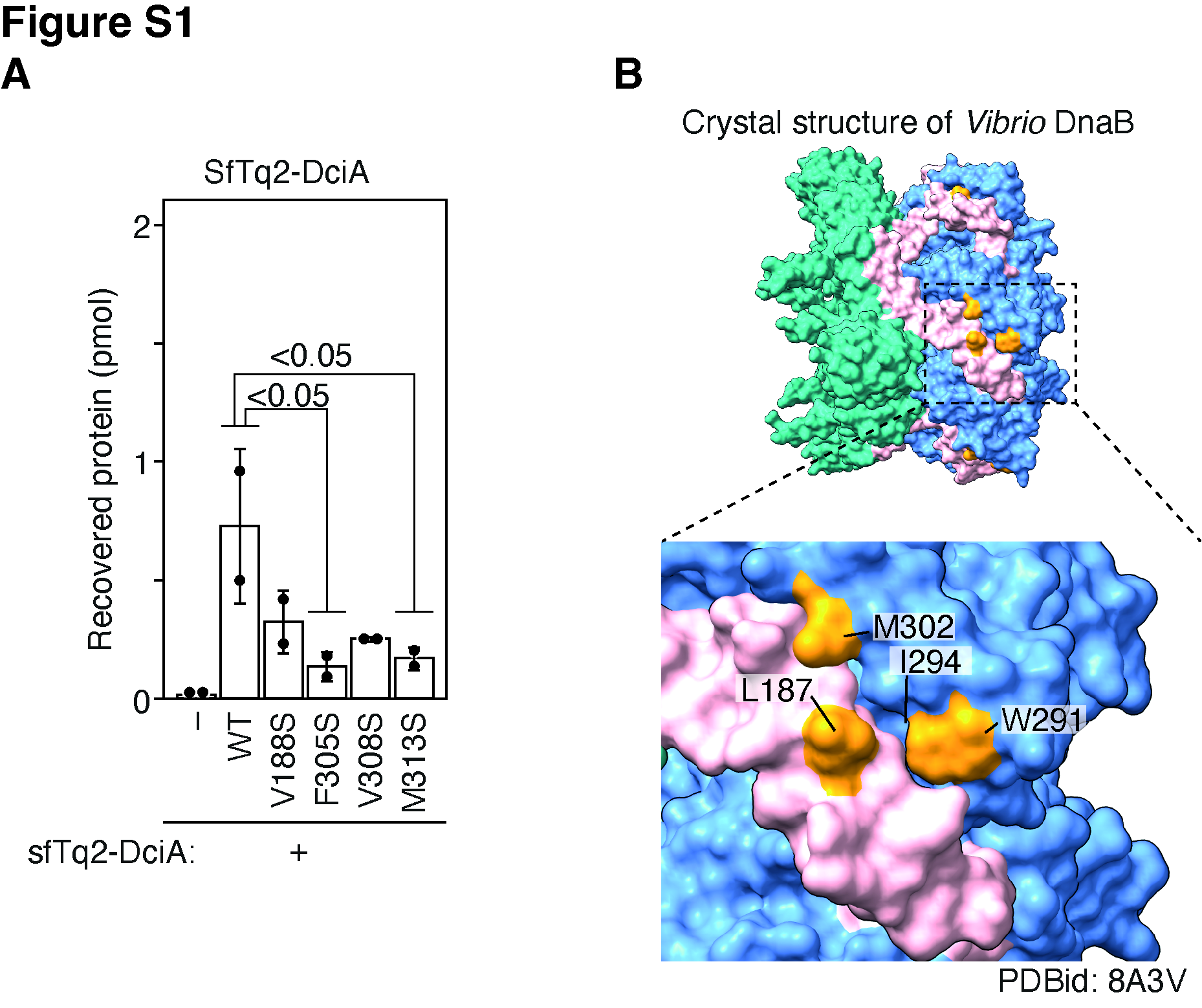


**Supplementary Figure S1 Pulldown assay**

(A) Recovery of sfTq2-DciA shown in *Figure 2FG* was plotted. The P value was calculated using Student’s t test.

(B) The crystal structure of *Vibrio* DnaB hexamer (PDBid: 8A3V) shown in surface representation. The three domains are colored as in *Figure 2B*. Residues corresponding to *cc*DnaB Val188, Phe305, Val308, and Met313 are highlighted in orange.


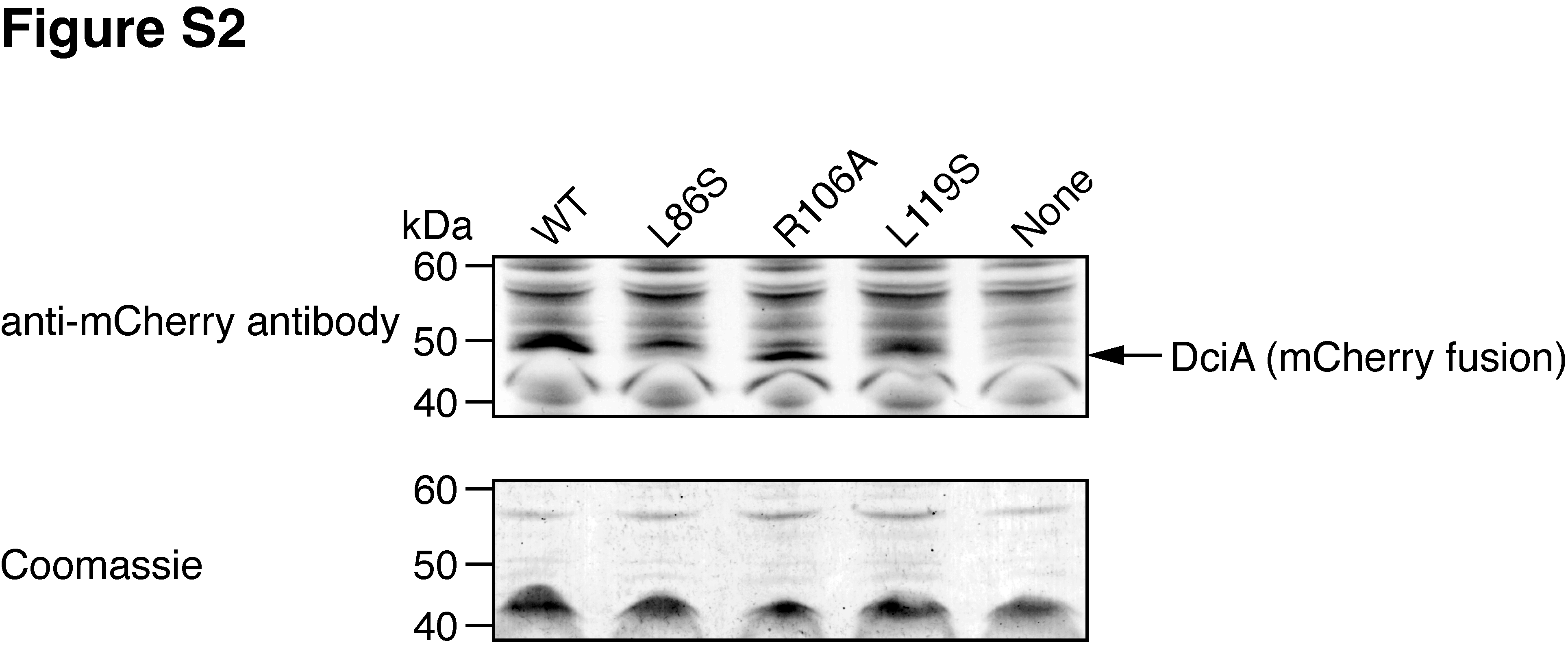


**Supplementary Figure S2 Protein stability of the DciA variants**

*C. crescentus* NA1000 cells bearing a pMR10 vector (none), pMR10-mChdciA (WT) or its derivatives pMR10-mChdciA(L86S), pMR10-mChdciA(R106A), or pMR10-mChdciA(L119S) were grown exponentially in PYE medium supplemented with kanamycin, followed by western blotting analysis using anti-mCherry antibody (1:1000). After a membrane transfer the polyacrylamide gel was stained with Coomassie brilliant blue as loading/transfer control.


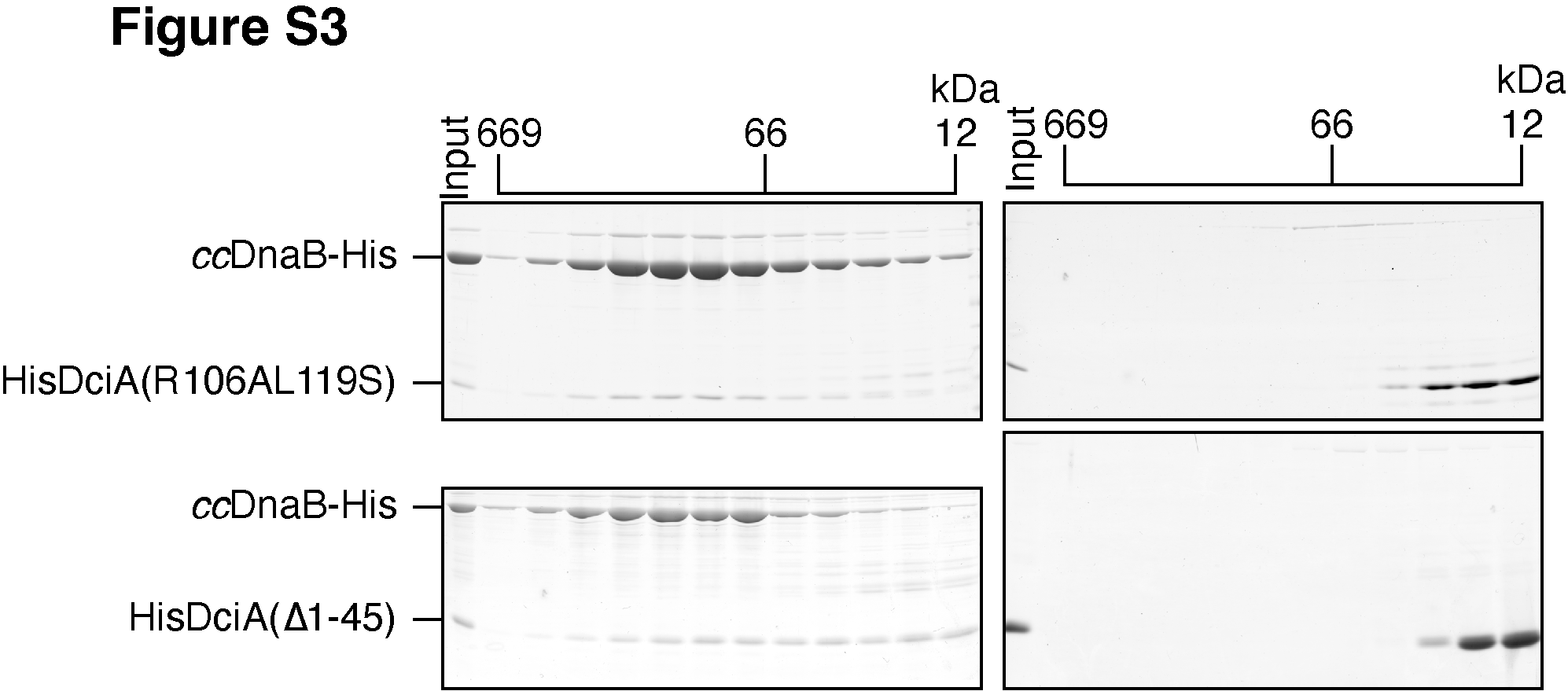


**Supplementary Figure S3 Size exclusion chromatography**

His-DciA(R106AL119S) and His-DciA(∆1-45) proteins were analyzed in the presence (*left*) or absence (*right*) of ccDnaB-His as described in the legend for *Figure 4A*.


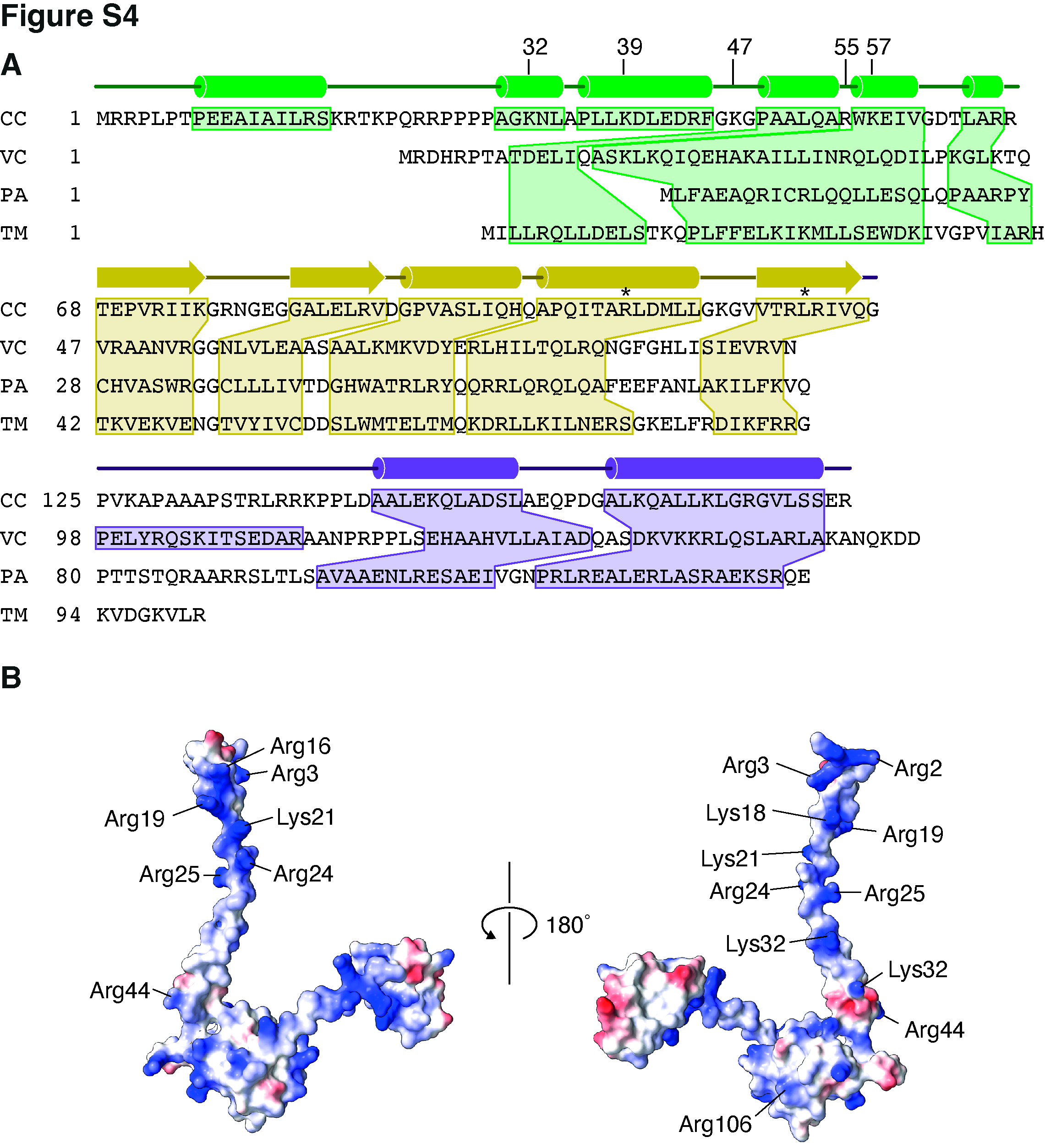


**Supplementary Figure S4 Comparison of the DciA family proteins**

(A) The AlphaFold3-predicted secondary structures of *C. crescentus* DciA (CCNA_00380; CC) and its homologs from *V. cholerae* (VC_2395; VC) *Pseudomonas aeruginosa* (PA4405; PA), and T. maritima (TM0832; TM) are aligned. Predicted α-helix and β-sheet are illustrated as in *Figure 3*.

(B) Detailed view of *Figure 5I*. Basic residues (Arg and Lys) spanning amino acids 1-45 of *C. crescentus* DciA are indicated.
